# The Role for Myc in Coordinating Glycolysis, Oxidative Phosphorylation, Glutaminolysis, and Fatty Acid Metabolism in Normal and Neoplastic Tissues

**DOI:** 10.3389/fendo.2018.00129

**Published:** 2018-04-12

**Authors:** Eric S. Goetzman, Edward V. Prochownik

**Affiliations:** ^1^Division of Medical Genetics, Children’s Hospital of Pittsburgh of UPMC, Pittsburgh, PA, United States; ^2^Division of Hematology/Oncology, Children’s Hospital of Pittsburgh of UPMC, Pittsburgh, PA, United States; ^3^Department of Microbiology and Molecular Genetics, University of Pittsburgh Medical Center, Pittsburgh, PA, United States; ^4^University of Pittsburgh Hillman Cancer Center, Pittsburgh, PA, United States

**Keywords:** fatty acid oxidation, glutaminolysis, glycolysis, mitochondria, oxidative phosphorylation, Randle cycle, Warburg effect

## Abstract

That cancer cells show patterns of metabolism different from normal cells has been known for over 50 years. Yet, it is only in the past decade or so that an appreciation of the benefits of these changes has begun to emerge. Altered cancer cell metabolism was initially attributed to defective mitochondria. However, we now realize that most cancers do not have mitochondrial mutations and that normal cells can transiently adopt cancer-like metabolism during periods of rapid proliferation. Indeed, an encompassing, albeit somewhat simplified, conceptual framework to explain both normal and cancer cell metabolism rests on several simple premises. First, the metabolic pathways used by cancer cells and their normal counterparts are the same. Second, normal quiescent cells use their metabolic pathways and the energy they generate largely to maintain cellular health and organelle turnover and, in some cases, to provide secreted products necessary for the survival of the intact organism. By contrast, undifferentiated cancer cells minimize the latter functions and devote their energy to producing the anabolic substrates necessary to maintain high rates of unremitting cellular proliferation. Third, as a result of the uncontrolled proliferation of cancer cells, a larger fraction of the metabolic intermediates normally used by quiescent cells purely as a source of energy are instead channeled into competing proliferation-focused and energy-consuming anabolic pathways. Fourth, cancer cell clones with the most plastic and rapidly adaptable metabolism will eventually outcompete their less well-adapted brethren during tumor progression and evolution. This attribute becomes increasingly important as tumors grow and as their individual cells compete in a constantly changing and inimical environment marked by nutrient, oxygen, and growth factor deficits. Here, we review some of the metabolic pathways whose importance has gained center stage for tumor growth, particularly those under the control of the c-Myc (Myc) oncoprotein. We discuss how these pathways differ functionally between quiescent and proliferating normal cells, how they are kidnapped and corrupted during the course of transformation, and consider potential therapeutic strategies that take advantage of common features of neoplastic and metabolic disorders.

## The Abnormal Metabolism of Cancer Cells: Glycolysis Versus Oxidative Phosphorylation (OXPHOS) and Beyond

The distinct metabolic behaviors of cancer cells have been appreciated since the 1950s when Otto Warburg first observed their high rates of glycolysis even when there was sufficient oxygen present to support OXPHOS (1–4). Such “aerobic glycolysis,” now termed the Warburg effect, was initially attributed to defective mitochondria but is now known to occur in rapidly growing normal cells and in cancers with no identifiable mutations in genes encoding mitochondrial proteins. More recently, the reprogramming of glutamine and fatty acid metabolism has also been identified in cancer cells (5–10). The still evolving consensus formulated over the past several years is that the altered metabolism of cancer cells is one of their so-called “hallmark” characteristics (11) and is both a direct and indirect consequence of oncogene and tumor suppressor gene mis-expression and/or mutation. Much less commonly does reprogramming occur as the result of metabolic gene mutation (12, 13). The two main advantages that metabolic re-wiring imparts to cancer cells are the ability to ensure sustained supplies of anabolic building blocks and to generate the energy needed for their assembly into macromolecules. This supports several other cancer hallmarks, including survival, sustained proliferation, tissue invasion and metastasis, and the participation in tumor-initiated angiogenesis (11).

Several general themes have begun to emerge from the study and cataloging of tumor-specific metabolic changes. One of these is that normal and malignant cells typically use the same basic metabolic pathways, which are deregulated in the latter and thus run at markedly different rates and/or are utilized to achieve different ends. For example, normal quiescent cells utilize glycolysis predominantly to generate small amounts of ATP (2 molecules/molecule of glucose) and pyruvate. Pyruvate is then completely oxidized by the TCA cycle within mitochondria to generate the reducing equivalents needed to power the electron transport chain (ETC) and to generate considerably more ATP (~36 additional molecules). By contrast, cancer cells often utilize glycolysis at an exaggerated pace for the same energy-generating purpose but also as a source of anabolic precursors to support rapid proliferation. For example, pyruvate is the initial substrate for the biosynthesis of alanine, aspartate, and threonine, and pyruvate’s immediate upstream precursor, phosphoenol pyruvate (PEP), is the starting substrate for tyrosine, tryptophan, and phenylalanine. The even more proximal glycolytic intermediate 3-phosphoglycerate can be directed into the synthesis of glycine and serine as well as purine nucleotides and the initial product of glucose catabolism, glucose-6-phosphate, can be diverted into the anabolic pentose phosphate pathway (PPP). TCA cycle intermediates such as citrate, succinyl coenzyme A (CoA), and oxaloacetate may also be used in non-mitochondrial biosynthetic pathways to furnish additional anabolic substrates for lipid, amino acid, and nucleotide biosynthesis, respectively. Any resulting depletion of these substrates from their mitochondrial stores may then be addressed by mobilizing the so-called anaplerotic (or “filling in”) reactions such as the conversion of glutamine to α-ketoglutarate, the β-oxidation of odd-chain fatty acids to succinyl-CoA, and the carboxylation of pyruvate to oxaloacetate.

Another theme is that these metabolic pathways are highly flexible and responsive in ways that ultimately benefit the growth and survival of the transformed cell. Indeed, cells with the most adaptable pathways will eventually outcompete their more metabolically rigid peers and be favored to survive and clonally expand over the course of tumor evolution. Such metabolic plasticity is particularly advantageous given the rapidity and extent to which the tumor microenvironment can change and the relatively small distances over which these changes can occur (14–17). The consequences of an inimical metabolic environment, which normally might promote cell cycle arrest or death, might be further assuaged by virtue of the loss of proapoptotic pathways mediated by TP53 and other tumor suppressors. In some cases, these losses not only delay or inhibit the apoptotic response to nutrient deprivation or the reactive oxygen species (ROS) associated with them but can themselves further alter metabolic pathways in favor of survival (18, 19). Maximizing survival and proliferation as a consequence of metabolic adaptability can also allow for the acquisition of additional mutations that further contribute to tumor evolution and adaptability (20).

Finally, a third theme is that some cancer-related metabolic reprogramming generates metabolites that can dramatically impact tumor behavior and even alter gene expression profiles. These effects can be direct or indirect, and the metabolites can either be the normal products of cellular respiration or the so-called “onco-metabolites,” which possess neomorphic properties and are generated as a consequence of mutations in mitochondrial enzymes. Examples of the first type include the excessive lactate generated by high rates of Warburg-type glycolysis. Lactate excretion lowers extracellular pH, thereby potentiating certain extracellular proteases and thus facilitating tumor invasiveness and metastatic spread (21). Lactate also upregulates vascular endothelial growth factor and hypoxia-inducible factor 1 alpha (HIF-1α), an oxygen-sensitive transcription factor that positively regulates glycolysis, particularly in collaboration with c-Myc (Myc), which is deregulated in the majority of human cancers (22–26). Furthermore, lactate, as well as Myc, can impart radioresistance in some tumors and contribute to the escape from immune surveillance (27–30). Tumor-generated lactate can also stimulate neighboring fibroblasts to increase their synthesis and release of hyaluronan, an extracellular high-molecular weight glycosaminoglycan, which increases motility and facilitates tumor cell spread (31). Point mutations in the TCA cycle enzymes isocitrate dehydrogenase (IDH) 1 or IDH2, most of which have been described in myeloid leukemias and gliomas (32–35), can cause the enzymes to generate the novel onco-metabolite d-R-2-hydroxyglutarate (d-R-2HG) rather than the normal TCA cycle intermediate α-ketoglutarate. d-R-2HG is a potent inhibitor of the Ten–Eleven Translocation 2 protein that normally converts 5-methylcytosine to 5-hydroxymethylcytosine, a reaction that serves as an intermediate step in DNA de-methylation (32, 36, 37). In a variation of this theme, both hypoxia- and lactate-mediated intracellular acidification can impart new catalytic properties to the enzymes lactate dehydrogenase (LDH) and malate dehydrogenase, allowing them to switch their normal substrate preferences and instead convert α-ketoglutarate to a 2HG enantiomer, L-S-2HG, which is also a potent epigenetic regulator (38–40). Finally, in an example that combines each of the above mechanisms, the accumulation of succinate, due to inactivating mutations in any one of the four subunits of the heterotetrameric succinate dehydrogenase (SDH) complex, has been linked to paragangliomas, pheocytochromocytomas, and gastrointestinal stromal cell tumors. While the exact mechanism by which excess succinate leads to transformation and why it is only associated with these rare tumor types are currently unknown, suspected culprits include excess ROS, HIF-1α stabilization, aberrant genome methylation, and tumor-promoting inflammatory changes (41–43).

Here, we review some of the major metabolic pathways that go awry in cancer, particularly those under the purview of Myc, and attempt to relate these to normal metabolic functions. It is important to emphasize that our focus on Myc arises from the fact that it is among the most frequently deregulated oncoproteins across all cancer types, is virtually never mutated, and regulates numerous metabolic functions (5, 22, 24, 44–46). Thus, metabolic alterations attributable to Myc are due to quantitative and not qualitative differences in its behavior, thus making it somewhat easier to understand its role in normal metabolic processes. Myc’s wide-spread overexpression in cancer can most likely be attributed to the fact that it is a major transcriptional integrator of most, if not all, normal and oncogenic growth factor pathways (22, 24, 44–47). Understanding how Myc reprograms metabolic pathways can explain much of how they are altered by upstream mutant oncoproteins that constitutively upregulate Myc expression. Moreover, the most prominent transcript families under Myc’s control tend to encode proteins that supervise energy production, anabolic pathways, protein synthesis, and cell cycle progression, all of which intimately impact both tumor and normal cell growth and survival and likely explain why many tumors are “addicted” to Myc (22, 45, 47–50). The differential regulation of these pathways by Myc permits unique glimpses into how they respond to different levels of this central transcriptional regulator while providing a basis for understanding why pharmacologic inhibition of Myc is considered a “Holy Grail” in cancer therapy and why it may also be useful in the treatment of non-malignant diseases of excessive cell proliferation (51, 52). We also summarize how certain pathways under Myc’s influence differ functionally in quiescent and proliferating normal cells and how they are altered in tumors by Myc’s deregulated expression (22, 24, 44).

## The Early Days: Hints That MYC (and Other Oncogenes) Regulate Cellular Metabolism

In the aftermath of the initial discovery that Myc is the cellular homolog of the retroviral v-Myc oncogene (53), little more than a year elapsed before recognizing that the former was commonly rearranged, amplified, and/or otherwise deregulated in human cancers, most notably Burkitt’s lymphoma (54–62). Shortly thereafter, endogenous Myc was found to be responsive to various mitogenic and differentiation-promoting stimuli, with the first type tending to upregulate and the second tending to downregulate its expression. Deliberately overriding these behaviors tended to reverse these tendencies, thereby demonstrating that Myc served as an active participant rather than a passive bystander. It was also shown that elevated and deregulated Myc expression frequently accompanies tumor progression and that the overexpression of Myc, either alone or in combination with other oncoproteins, was potently transforming both *in vitro* and *in vivo* (63–88).

It was not until the mid-1980s, however, that the relationships between protooncogene expression, normal and neoplastic proliferation, and altered metabolism began to truly take shape and mold our current outlook. For example, the eventual classification of Myc as a so-called “immediate-early” gene in response to growth factor stimulation in fibroblasts (65, 78, 80, 81) led to the finding that the ectopic conditional expression of Myc alone was sufficient to promote an abortive G0 → S-phase transition (63). Shortly thereafter, studies in quiescent thymocytes and fibroblasts additionally showed that Myc induction following mitogenic stimulation was preceded by rapid and sequential changes in phosphoinositide metabolism, Ca^2+^ release, the activation of phospholipid-dependent kinase C and altered Na^+^/H^+^ exchange (89, 90). Enforced Myc or Ras expression in log-phase Rat1 fibroblasts was also then found to stimulate glycolysis, which was further enhanced by the addition of the growth factor TGF-β (91). Subsequently, differential screening of cDNA libraries prepared from quiescent and serum-stimulated Balb/3T3 murine fibroblasts identified a small number of transcripts that were induced within 12 h of applying this mitogenic stimulus (92). In addition to Myc, these encoded LDH and enolase, thus hinting at the idea that Myc might be involved in the regulation of metabolism, that specific genes within the glycolytic pathway might be important for initiating the biomass accretion necessary for growth and division, and that Myc might somehow be involved in the regulation of these genes. Being mindful of proper historical context, it is important to note that these studies preceded by over a year the initial reports that Myc was a DNA-binding transcription factor (93–96). Thus, the relationship between Myc and transcripts encoding metabolic enzymes remained enigmatic until this critical Myc function was unmasked. It is now known that nearly all genes encoding glycolytic enzymes are direct Myc targets and that the Warburg effect is at least partially under Myc control (97–99). Together, these findings underscore two of the three major themes mentioned in the preceding section: first, that the metabolic changes accompanying rapid normal and malignant proliferation utilize the same pathways as normal quiescent cells, although not always for the same reasons; and second, that malignant cells maintain or corrupt these pathways for the singular purpose of gaining a proliferative and/or survival advantage over their normal counterparts, or even their transformed but less metabolically adaptable relatives.

Over the next several years, it gradually emerged that one of Myc’s principle functions, both in normal and cancer cells, was to regulate cell mass and, in doing so, to directly modulate the expression of genes involved in ribosomal biogenesis including ribosomal structural genes, tRNAs, rRNAs, and all three eukaryotic RNA polymerases that control the expression of these genes (100–109). The identification of these novel Myc targets was a satisfying observation as it began to shed light on how an oncoprotein could promote proliferation on the one hand while coordinating this with protein synthetic rates (or at least the protein synthetic machinery) and the doubling of cell mass that must precede division on the other (110). It also complemented earlier observations that transformed cells increase their uptake of both amino acids and the glucose analog 2-deoxy-glucose (2-DG) (91, 110). Yet even by this time and with these observations in hand, little attention was paid to the other metabolic changes needed to support the growth and proliferation of cancer cells. Not that they had been entirely ignored; indeed, there were hints as early as the mid-1950s that such changes were intimately associated with the increased proliferation of cancer cells including the eponymous Warburg effect mentioned earlier (3, 111, 112).

## MYC and the Regulation of Glycolysis, OXPHOS, and Energy Balance

**Figure 1:**
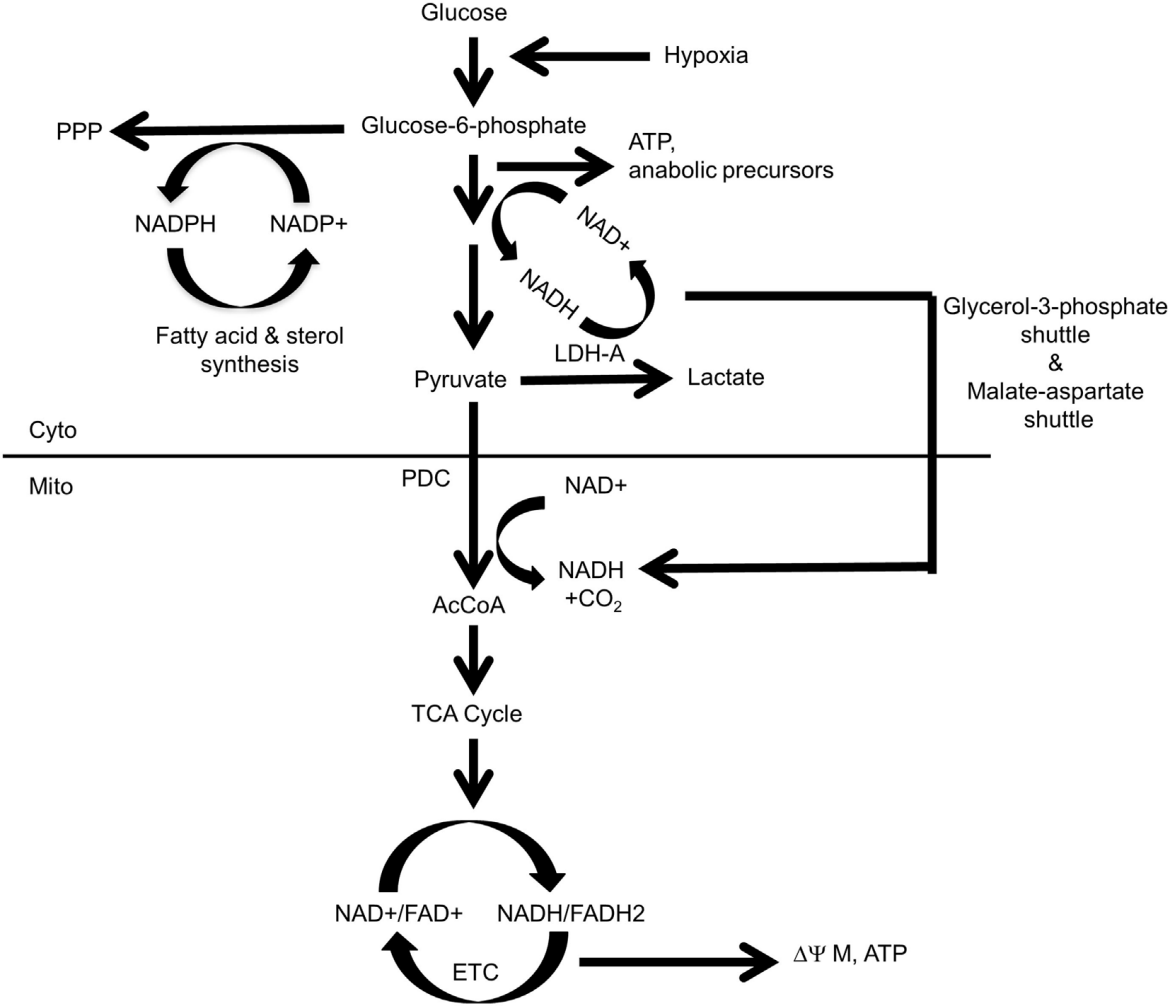
Control and distribution of ATP and reducing equivalents derived from glycolysis and oxidative phosphorylation (OXPHOS). Under normal aerobic conditions, most glycolysis-derived pyruvate produced by quiescent or slowly growing cells is converted to acetyl-CoA (AcCoA) in the mitochondria, with little being converted into lactate. This provides the mitochondrial reducing equivalents, in the form of NADH and FADH2, to power the electron transport chain (ETC), to generate a proton gradient (ΔΨM) and to establish the protomotive force necessary to drive ATP generation *via* Complex V. Excess cytoplasmic reducing equivalents in the form of NADH may be transferred to the mitochondria *via* the glycerol-3-phosphate and malate–aspartate shuttles and can potentially support OXPHOS when AcCoA levels are low. During hypoxia, glucose uptake, glycolytically derived ATP synthesis, and the “A” isoform of LDH (LDH-A)-catalyzed conversion of pyruvate to lactate are accelerated due to an inability of the ETC to reduce molecular oxygen to water and generate TCA cycle-derived ATP. Most pyruvate will be converted to lactate, thereby generating more NAD^+^ and sustaining anaerobic glycolysis. This positive feedback loop ensures a constant and balanced source of ATP as an energy source and the regeneration of NAD^+^ to serve as an electron acceptor for continued glycolysis. By contrast, dividing cells have “divided loyalties” with regard to the disposition of glycolytic intermediates, with some being used to generate pyruvate and others being used as anabolic building blocks. Just as glycolysis and lactate production engage in a positive feedback loop to provide a balanced amount of NAD^+^ and NADH in support of one another’s activity, the pentose phosphate pathway (PPP) and the biosynthesis of fatty acids and sterols, generate NADPH and NADP^+^, respectively, to support each others’ pathways. The pyruvate dehydrogenase complex (PDC), which includes pyruvate dehydrogenase, is the key enzymatic complex that connects glycolysis to the TCA cycle. It catalyzes the conversion of the last product of glycolysis, pyruvate, to the entry-level substrate for the TCA cycle, AcCoA. In doing so, it contributes one-fourth of the NADH that is produced by a single round of the TCA cycle.

As emphasized above, Myc’s pivotal role in the control of carbohydrate metabolism emerged gradually in the mid-late 1980s and early 1990s. However, it first required demonstrating that Myc is a sequence-specific DNA-binding transcription factor (92–95, 113, 114), together with the subsequent development of high-throughput and unbiased methodologies to identify Myc-target genes (115–119). From among the earliest such attempts emerged one of the first direct Myc-target genes (120), namely, the “A” isoform of LDH (LDH-A), which, as noted above, had been previously identified, along with Myc itself, as being induced by serum in fibroblasts (92). Shim et al. and Lewis et al. subsequently demonstrated that Myc-mediated fibroblast transformation was attenuated following the genetic suppression of LDH-A, that these fibroblasts were highly susceptible to apoptotic death in the face of glucose deprivation and that LDH-A could cooperate with another Myc target, rcl, to transform fibroblasts (120–122). Rather than being directly transforming, however, it seems more likely that LDH’s role is more related to the fact that the LDH-mediated generation of lactate from pyruvate requires NADH as an electron donor and that the product of this reaction, NAD^+^, may then function as an electron acceptor to support more proximal glycolytic reactions (Figure 1). This co-dependency between glycolysis and lactate generation ensures that the former pathway can be efficiently maintained regardless of its rate, particularly when oxygen concentrations are low, the generation of lactate is high and glycolysis is the major ATP source. Further supporting the importance of this positive feedback loop was the subsequent observation that most glycolytic enzyme-encoding genes are regulated at some level by Myc (120, 123–127). Thus, the production and excretion of lactate, an otherwise energetically wasteful activity, is actually necessary to sustain the Warburg effect as it ensures the continuous generation of NAD^+^ to serve as an electron acceptor during glucose oxidation. In the presence of oxygen, cytoplasmic NAD^+^ may also be supplied *via* the glycerol-3-phosphate shuttle and the malate–aspartate shuttle in which reducing equivalents are transferred to the mitochondria in exchange for generating an oxidized cytoplasm to maintain glycolysis (128, 129). While these shuttles are likely to be less important under the hypoxic conditions that often prevail during tumor growth, they nonetheless allow for cross talk, cooperation, and coordination between glycolysis and OXPHOS in well-oxygenated environments (130–132).

Warburg-type glycolysis, in conjunction with the abovementioned shuttles, couples the generation of reducing equivalents in the form of NADH with their indirect transfer into the mitochondrial matrix to drive the ETC and maintain membrane potential (ΔΨM) and the protomotive force that generates ATP *via* Complex V. The glycerol-3-phosphate and malate–aspartate shuttles thus provide sources of mitochondrial reducing equivalents independent of those generated by the TCA cycle when the supply of pyruvate-derived acetyl-CoA (AcCoA) might be compromised due to the diversion of glycolytic intermediates for anabolic purposes and/or the production of lactate. These shuttles also sustain mitochondrial NADH levels when TCA-generated substrates are consumed by other anabolic reactions, such as the synthesis of lipids and certain amino acids. Interestingly, rather than being transferred to Complex I or Complex II as occurs with TCA cycle-derived reducing equivalents, the NADH supplied by these shuttles is confined to the inner mitochondrial membrane and surrenders its electrons directly to Coenzyme Q and then to Complex III of the ETC (133). Such reducing equivalents, derived from glycolysis, thus provide an electron source that bypasses Complex I and Complex II and is under different regulatory supervision.

Glycolysis also indirectly generates reducing equivalents in the form of NADPH during the proximal steps of the PPP. NADPH is a source of cytoplasmic reducing equivalents that supports the reductive *de novo* synthesis of sterols and fatty acids, regenerating NADP^+^ in the process and providing an energetic and self-sustaining link between the biosynthesis of pentose sugars and lipids (134, 135). Thus, high rates of glycolysis and the lactate generation made in response to oncogenic stimuli such as Myc ensure a well-balanced and oxygen-independent supply of cytoplasmically generated ATP, reducing equivalents, and critical anabolic precursors (Figure 1).

Increased glycolytic flux ensures a rich supply of substrates that can be redirected into energy-consuming anabolic pathways with sufficient amounts remaining to generate AcCoA and drive the TCA cycle. But precisely how this occurs and whether any control is exerted remains somewhat enigmatic. Do the glycolysis-linked pathways simply “drink from the fire hose” of glycolytic intermediates as they rush down the pathway, or are there mechanisms that specifically direct glycolytic intermediates into anabolic pathways at the expense of mitochondrial OXPHOS, aided by the law of mass action? Support for the latter notion comes with the observation that the expression of the two isoforms of pyruvate kinase (PK), PKM1, and PK2, is altered in some cancers. PKM1, which tends to predominate in normal tissues, is replaced by PKM2, which has a higher *K*_m_ for its substrate, PEP; the *K*_m_ is even further increased by posttranslational modification. The overall lessened PK activity serves to redirect PEP and other upstream glycolytic substrates away from energy-generating OXPHOS and into energy-consuming anabolic pathways (136–140).

The PKM1 and PKM2 isoforms differ from one another as a result of mutually exclusive alternate mRNA splicing such that the PKM1 transcript encodes exon 9 but not exon 10 and PKM2 encodes exon 10 but not exon 9. The splicing decision is governed by at least three heterogeneous nuclear ribonucleoproteins, hnRNP1, hnRNP2, and hnRNP, which bind to intron regions flanking exon 9 and negatively regulate its splicing. Interestingly, hnRNP1 and hnRNP2 are positively regulated by Myc (141, 142). However, the switch to aerobic glycolysis, by whatever means, hardly signals an irreversible commitment; indeed, not only is the Warburg effect plastic but many tumors-even those of similar types are more dependent upon OXPHOS than glycolysis for their energy requirements and/or can rapidly balance these two processes to suit their needs (143). In addition to regulating glycolysis, Myc also exerts significant influence over mitochondrial structure and function. Initially relegated to a metabolic backwater following the realization that many cancer cells rely on the Warburg effect at the expense of OXPHOS, mitochondria do in fact play important roles in cancer metabolism (144, 145). Indeed, certain cancers remain highly reliant on OXPHOS as a means of energy production and show little predilection for Warburg-type metabolism (146). Analogous to glycolysis, however, both mitochondria and the energy producing pathways they encompass undergo significant structural and functional revisions in response to Myc deregulation.

Among the first studies to examine Myc’s effect on mitochondrial structure and function were those of Li et al. (147). Utilizing P493-6 human B cells transfected with a tetracycline-inducible human Myc transgene, they showed that mitochondrial mass increased approximately twofold within 72 h of Myc induction, as measured by nonyl acridine orange staining, MitoTracker staining and mitochondrial DNA (mtDNA) content. Oxygen consumption increased to a similar extent thereby indicating a close relationship between structure and function. Transcriptional profiling also identified approximately 200 Myc-responsive genes encoding mitochondrial proteins, most of which were upregulated. Most notable among these was the transcript encoding Tfam, a nuclear-encoded mitochondrial transcription factor that also participates in mtDNA replication (148–150). The *TFAM* gene proximal promoter was also shown to contain a Myc-binding E-Box element. In serum-stimulated fibroblasts, endogenous Myc was shown to bind the same site (147). Although no functional studies were performed, it was assumed that this positive effect of Myc on mitochondrial biogenesis was accompanied by a parallel increase in TCA cycle activity and OXPHOS.

Elucidating the role of endogenous Myc in the regulation of metabolic pathways was hampered for quite some time by the lack of a suitable knockout model. This is because even short-term Myc depletion in virtually all cell lines is accompanied by cell cycle arrest, apoptosis, or differentiation, thereby severely compromising long-term studies (50, 151, 152). Similarly, whole body inactivation of the *myc* gene is an embryonic lethal (153). Thus, the generation of a *myc^−/−^* cell line from rat fibroblasts by Mateyak et al. in 1997 (154) provided a tremendous technical advance despite the fact that it remains unresolved as to how these cells survive and replicate.

Although viable, the *myc^−/−^* fibroblasts described by Mateyak et al. (154) are extremely abnormal. For example, they divide only once every 2–3 days versus every 18–24 h for the *myc^+/+^* parental line, display an extremely flattened morphology and are highly contact inhibited. Cell cycle regulation is severely compromised at multiple points, with *myc^−/−^* cells showing a *ca*. 12-fold reduction in the expression of CyclinD–Cdk4 and CyclinD–Cdk6 complexes during the G_0_/G_1_ transition and delayed activation of CyclinE–Cdk2 and CyclinA–Cdk2 complexes (145, 155). That most of these abnormalities can be rescued at least partially with retrovirally expressed Myc, the Myc homologs N-Myc and L-Myc, or the Myc-target genes MYCT1/MT-MC1, HMG-IY and SHMT (156, 157) indicates that the various phenotypes of *myc^−/−^* fibroblasts are directly related to *myc* gene inactivation and do not represent compensatory and Myc-independent growth-enhancing adaptations.

The absence of Myc was also reflected in the mitochondrial structure and metabolism of these cells. Graves et al. (158) showed that, compared with *myc^+/+^* fibroblasts, the mitochondria of *myc^−/−^* fibroblasts were smaller, fewer in number, deficient in cristae and poorly interconnected. The activation of a Myc-estrogen receptor fusion protein (MycER) by 4-hydroxytamoxifen (4OHT) complemented these defects, although, surprisingly, it required 4–5 weeks before maximal mitochondrial mass was restored. The extremely low ΔΨM of these cells (*myc^−/−^* MycER) cells also increased in parallel over this same time period. This provided indirect evidence that Myc was likely controlling some aspect of the TCA cycle and/or the availability of reducing equivalents needed to drive the ETC. Following 4OHT’s removal and the silencing of Myc, mitochondrial mass, ΔΨM and interconnectivity returned to near the baseline levels of *myc^−/−^* cells over another prolonged period exceeding 10 days (158).

In addition to the above major structural defects, which confirmed and extended those of Li et al. (147), mitochondrial function was also severely compromised in *myc^−/−^* cells in a manner that mirrored their structural defects. For example, the basal oxygen consumption rate (OCR) of *myc^−/−^* cells was about half that of *myc^+/+^* cells and only 15% that of *myc^−/−^* cells stably reconstituted with a lentiviral vector that drove high level, constitutive Myc expression (*myc^−/−^* Myc cells). The most prominent effect of Myc on OCR was seen in *myc^−/−^* Myc cells where the maximum respiratory capacity in response to the de-polarizing agent FCCP was >20 times higher than that of *myc^−/−^* cells and 6 times higher than that of *myc^+/+^* cells. Because Myc positively regulates virtually all glycolytic genes (96, 123, 124, 159), the basal rate of glycolysis in *myc^−/−^* cells was also about half that of *myc^+/+^* cells and one-third that of *myc^−/−^* Myc cells (158).

Consistent with their markedly impaired OXPHOS and glycolysis, *myc^−/−^* cells showed a nearly 70% reduction in basal ATP levels, which normalized following Myc re-expression. In all three cell lines (*myc^−/−^, myc^+/+^*, and *myc^−/−^* Myc), exposure to 2-DG caused a more pronounced ATP depletion than did the inhibition of OXPHOS with rotenone. Taken together, these results suggested that at least half the energy in these cells was derived from glycolysis. Consistent with their ATP deficient state, *myc^−/−^* cells expressed high levels of activated (phosphorylated) AMP-activated protein kinase (AMPK), a serine/threonine kinase that responds to ATP depletion (or more precisely to a high AMP:ATP ratio) by upregulating ATP-generating pathways and downregulating ATP-consuming pathways (160–162). However, since many of the energy-sparing and energy-generating effects of AMPK rely on the upregulation of Myc (160, 163, 164), AMPK seems to be unable to achieve a state of true energy equilibrium in *myc^−/−^* cells, thereby leading to its constitutive activation in the face of a chronic energy deficit. The restoration of Myc in *myc^−/−^* Myc cells did lead to AMPK dephosphorylation that correlated with the normalization of ATP levels (160).

The observation that *myc^+/+^* and *myc^−/−^* Myc cells contained identical ATP levels could not initially be reconciled with the finding that the latter cells had significantly higher rates of glycolysis and OXPHOS. This discrepancy was resolved by showing that the ATP half-life in the latter cells was nearly 50% shorter (2.6 versus 3.6 min) (158). This was consistent with the previous finding that *myc^−/−^* WT cells had significantly faster growth rates than *myc^+/+^* cells and thus likely utilized more ATP (165).

To further understand the role of endogenous Myc in maintaining basal rates of glycolysis and OXPHOS in transformed cells, Graves et al. (158) utilized the conditional, doxycycline-regulatable expression of a short hairpin RNA directed against Myc to silence the oncoprotein’s expression in A549 human small cell lung cancer cells, which normally express high levels of Myc. Conforming to the findings in the above-discussed rat fibroblast studies, the knockdown of Myc was associated with marked growth inhibition, a flattened cellular morphology, reduced mitochondrial mass, and the collapse of ΔΨM.

Relative to *myc^+/+^* and *myc^−/−^* Myc cells, *myc^−/−^* cells demonstrated abnormalities in overall structure and function of the ETC (158). Among these were reduced amounts of the so-called “supercomplexes” (SCs) between Complexes I, III, and IV, which allow for more efficient electron transfer (166, 167). Consistent with their atrophic mitochondrial cristae, which are believed to serve as a platform for the formation and accretion of SCs (168, 169), *myc^−/−^* mitochondria also contained lower levels of Complex I, II, and III as well as both the monomeric and dimeric forms of Complex V ATPase (V_m_ and V_d_, respectively) relative to *myc^+/+^* cells. In general, SC function in *myc^−/−^* cells, as measured by *in situ* enzymatic activity of individual complexes separated by non-denaturing blue native gel electrophoresis (BNGE), closely matched Coomassie Blue staining patterns. *myc^−/−^* fibroblast mitochondria also contained significant levels of an enzymatically inert complex (Complex “X”) that was shown by mass spectroscopy to be comprised of multiple subunits from Complexes II–V. It was speculated that during periods of relative oxidative quiescence, Complex X functions as a reservoir for certain mitochondria proteins, which can be rapidly summoned and assembled into their respective ETC complexes in response to increased metabolic needs. This seems like a logical cellular strategy in that cells with depleted energy levels as a result of ETC dysfunction might be better served by utilizing preexisting ETC components for rapid assembly and resumption of ETC function rather than expending even more energy by synthesizing them anew. On the other hand, the surprisingly long time it takes to restore normal mitochondrial structure and function in *myc^−/−^* cells (158) raises questions as to whether this is the true function of this complex.

Interestingly, *myc^−/−^* Myc cells showed only a partial normalization of ETC structure and function as measured by the above methods, despite their high-level Myc expression. Relative to *myc^+/+^* cells, *myc^−/−^* Myc cells contained only about half the levels of SCs, two-thirds the level Complex V monomers (V_m_) and ~15% the level of Complex V dimers (V_D_) as measured by both enzymatic activity and BNGE. While both V_D_ and V_M_ possess ATP synthase activity, the dimer appears to be more important for dictating the shape of mitochondrial cristae (170). Because the high-level re-expression of Myc also greatly increased glycolysis, it was surmised that this failure to entirely normalize ETC structure and function was due to a combination of factors including structural changes to the mitochondria and their cristae, differences in the relative contribution of glycolysis and OXPHOS to the energy landscape, subtle nuances relating to the control of Myc protein expression, and differential cellular growth rates and their resulting anabolic requirements (158).

The normalization of mitochondrial morphology by the enforced re-expression of Myc in *myc^−/−^* Myc cells (158) suggested that Myc might influence mitochondrial fusion and/or fission. The former process is regulated by the so-called “mitofusin” proteins such as Mfn1, Mfn2, and Opa1 whereas the latter process is regulated by the proteins Fis1 and Drp/Dlp (171–175). Fusion is believed to maximize mitochondrial energy production by allowing old and/or damaged organelles to be “rejuvenated” by combining their contents with those of younger ones, thereby extending their life span and functional integrity and capacity (176, 177). By contrast, fission provides a mechanism by which mitochondrial mass can be reduced during periods of relative metabolic quiescence or Warburg-type respiration or by which defective and/or aged mitochondria can be eliminated. Both fusion and fission can exert significant influence upon mitochondrial energy production and cell survival (176, 178, 179).

Virtually, all the above mitochondrial fission and fusion proteins were expressed at higher levels *in* Myc replete cells relative to *myc^−/−^* cells making it unclear how, as an integrated group, they affected mitochondrial biogenesis and, if so, which of the processes this favored. The question was answered by experiments in which 4OHT-treated *myc^−/−^* MycER cells were separately transfected with mitochondrially targeted green or red fluorescent proteins (GFP or RFP, respectively). Cells from the two populations were then mixed and fused by exposure to polyethylene glycol and the rate of GFP^+^ and RFP^+^ mitochondrial fusion into “yellow” merged organelles was quantified either in the continued presence of 4OHT or following its removal. Cells actively expressing Myc showed nearly twofold higher rates of mitochondrial fusion compared with cells in which Myc had been silenced for 2 days by removing 4OHT. The faster rate of mitochondrial turnover in the former cells suggested that they were under constant pressure to maintain only the youngest and healthiest mitochondria to meet the increased metabolic needs of this energetically more demanding and faster growing population. Thus, along with affecting the levels of key mitochondrial transcription factors such as Tfam (123, 147), Myc also influences mitochondrial biogenesis and lifespan by modulating the levels of fission/fusion proteins.

Subsequent work demonstrated that alterations in mitochondrial structure and function can reciprocally impact the function of both endogenous and overexpressed Myc. In these studies, Sarin et al. (180) enforced the expression of the mitochondrial fission protein Drp/Dlp in Rat1a-MycER cells, leading to a state of non-stop, fission-induced mitochondrial fragmentation and a pronounced reduction in overall mitochondrial size, mass and interconnectivity. Accompanying this was a nearly 15-fold higher rate of mitochondrial fusion relative to control cells suggesting that Drp1/Dlp1-overexpressing cells constantly upregulate fusion in a futile compensatory attempt to offset their excessive Drp1/Dlp1-driven fission. Despite the fact that these cells expressed normal levels of Myc, their mitochondria were both structurally and functionally reminiscent of those from *myc^−/−^* cells. Structurally, their ETC complexes were defective; BNGE revealed a 28% reduction Complex I, a 45% reduction in Complex V and a 38% reduction in SCs. Furthermore, Complex “X,” the proposed repository for certain ETC subunits in *myc^−/−^* cells (158), now appeared. Thus, enforced and uncorrected mitochondrial hyper fission leads to a loss of mitochondrial structural integrity resembling that of *myc^−/−^* cells in the face of otherwise normal Myc levels.

Further characterization of Drp1/Dlp1-overexpressing cells showed that, like *myc^−/−^* cells, they too had a critical energy shortage, with a >80% reduction in ATP levels and impaired glycolysis and OXPHOS. This energy-depleted state, coupled with the failure to adequately compensate for it, was evidenced by a *ca*. 30% decrease in mean cell volume and a >10-fold increase in phosphoAMPK (180). Collectively, these findings suggested that the price for such energy-conserving processes was a reduction in energetically demanding biomass accumulation. Most likely as a result of their abnormal ETC structure and/or their ATP deficit, Drp1/Dlp1 overexpressing cells, like *myc^−/−^* cells, expressed higher levels of ROS than control cells (181, 182). No obvious growth differences between control and Drp1/Dlp1-overexpressing cells were observed under standard conditions although the latter were significantly more resistant to apoptosis in response to Myc overexpression or serum deprivation and this was supported by the less pronounced release of cytochrome c from mitochondria. Treatment with 5-amino-1-b-d-ribofuranosyl-imidazole-4-carboxamide, an AMP analog that activates AMPK and increases ATP pools (161, 162, 183) doubled the ATP content of Drp1/Dlp1 overexpressing cells, normalized their size and increased their sensitivity to apoptotic stimuli (180). The prolonged survival of Drp1/Dlp1-overexpressing cells may reflect the fact that ATP depletion tends to protect against apoptosis, perhaps by inhibiting caspases 3 and 8 and Apaf-1, and that, in some circumstances AMPK activation can restore or promote apoptosis (184–188). Collectively, these results show that enforced mitochondrial fission driven by Drp1/Dlp1 can override Myc’s role in maintaining normal mitochondrial integrity and adequate ATP levels. Whether this is due to a lack of response of mitochondria as a result of their inability to fuse in response to Myc (as seems likely) or another effect of Drp1/Dlp1 remains to be determined.

**Figure 2:**
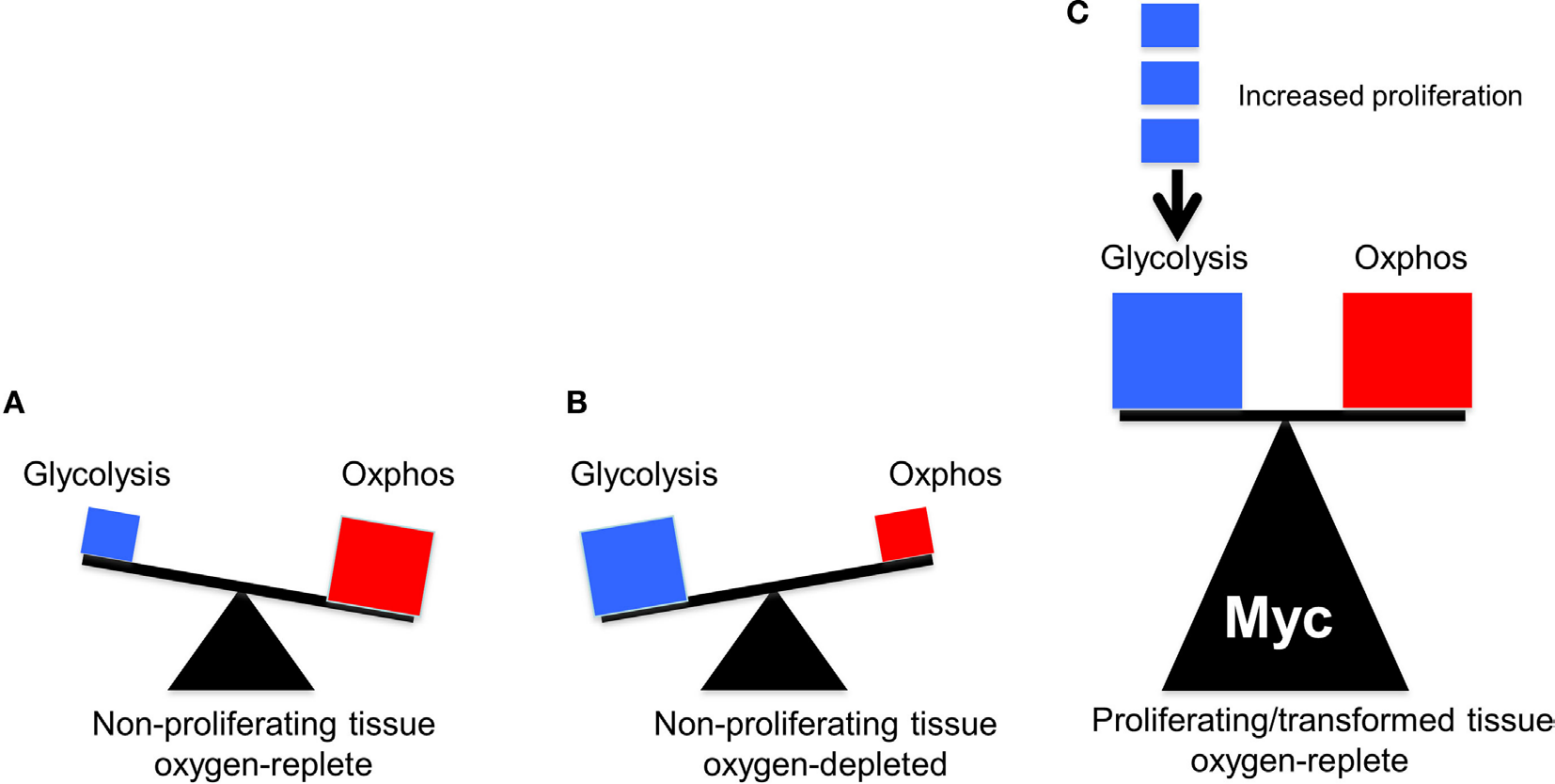
The balance between glycolysis and oxidative phosphorylation (OXPHOS) in normal and tumor cells. The sizes of the triangles are meant to indicate the relative metabolic activity under each of the depicted conditions. **(A)** Under aerobic conditions, glucose in quiescent, non-proliferating cells is converted in a linear fashion into pyruvate, which is then consumed by the TCA cycle to produce the bulk of ATP (Figure 1). **(B)** Under hypoxic periods in these same cells, OXPHOS is no longer able to furnish ATP. Energy production then switches rapidly to glycolysis, which can temporarily maintain energy supplies. Both glucose uptake and glycolysis can be highly upregulated although at the expense of generating considerable amounts of lactate. **(C)** In well-oxygenated, rapidly proliferating tumor cells and normal cells, glycolysis and OXPHOS are both upregulated and balanced so as to provide for the most efficient production of ATP, the most rapid biomass accumulation and the most rapid rate of proliferation. The metabolic building blocks for these processes are derived from both the glycolytic pathway and the TCA cycle, thus explaining the need for each. Examples of the first include the diversion of glucose-6-phosphate into the pentose phosphate pathway to provide pentose sugars for nucleotide synthesis (Figure 1), the diversion of 3-phosphoglycerate for the synthesis of purine nucleotides and the diversion of phosphoenol pyruvate for the synthesis of tyrosine, tryptophan, and phenylalanine. Examples of the second include the transport of citrate into the cytoplasm for use in fatty acid and sterol biosynthesis and the diversion of oxaloacetate into the cytoplasm for conversion in aspartate, asparagine, and pyrimidine nucleotides. While the graphic here depicts an equal contribution by both glycolysis and OXPHOS, their relative contribution to overall metabolism can shift in response to proliferative rates (indicated by the small blue boxes), oxygen tension, and the supply of environmentally derived nutrients. In rare cancers, mutations in select TCA cycle genes can impart increased, and less reversible, reliance on aerobic glycolysis as a means of generating energy, thus confirming to Warburg’s original explanation of his eponymous effect (41–43).

The above studies showed that, at least in proliferating fibroblasts propagated *in vitro*, where nutrient supplies and oxygen concentrations are high and non-rate-limiting, both glycolysis and OXPHOS are subject to positive regulation by endogenous Myc. Moreover, they establish that the overexpression of Myc, to the levels required to drive proliferation and transformation, continues to exert a simultaneous positive effect on both glycolysis and OXPHOS. The effects are quite heterogeneous, are both direct and indirect and involve changes in transcripts encoding glycolytic enzymes and mitochondrial structural and functional components. These studies show that the Warburg effect and OXPHOS are by no means mutually exclusive. Rather they are better viewed as being complementary, with neither one being entirely dispensable (Figure 2).

The role of endogenous Myc in sustaining glycolysis and mitochondrial function has received additional support from studies in other cell types with several structurally and mechanistically distinct small molecule Myc inhibitors (189–191). HL60 promyelocytic cells exposed for only 2–5 days to these compounds dramatically reduced their ATP content, activated AMPK, accumulated neutral lipids and downregulated Myc as they underwent terminal myeloid differentiation. Because the manipulation of Myc had long been know to exert profound effects on the differentiation of hematopoietic and other cell types (68, 72, 77, 87, 192), Wang et al. repeated these experiments using two mechanistically distinct inhibitors of Complex I, metformin and rotenone (191). Decreased ATP, AMPK activation and myeloid differentiation were again noted, but Myc levels were unaffected. These studies supported the idea that ATP levels are a strong and Myc-independent determinant of differentiation, at least in myeloid cells (193). They further implied that a major role of Myc in differentiation is to maintain ATP levels, most likely with the purpose of allowing for the continued accumulation of biomass. This is also likely aided by Myc’s ability to induce the expression of many genes involved in cell cycle progression (101, 194, 195). Differentiation may therefore represent one possible means of maintaining viability in response to energy-depleted states.

In other studies using a transgenic mouse model of neuroblastoma driven by the highly related Myc homolog N-Myc, Zirath et al. (196) showed that the treatment of tumor-bearing animals with the small molecule Myc inhibitor 10058-F4 (197), which also binds to and distorts the structure of N-Myc (198), inhibited tumor growth and promoted tumor differentiation as evidenced by neurite outgrowth. It also caused the accumulation of high levels of intracellular neutral lipid (196).

Activated T-cells are among the most rapidly dividing metazoan cells. Following antigen stimulation, they accumulate biomass for approximately 24 h and then enter a phase of rapid proliferation and clonal expansion, with cell division occurring as frequently as every 4 h (199). It has long been known that this replicative phase is associated with markedly increased glycolysis and glutaminolysis although the precise pathways needed to effect this metabolic reprogramming remain ill defined (200–202). Wang et al. (203) investigated Myc’s contribution to the biomass accretion and proliferative expansion following *ex vivo* stimulation of murine T-cells with anti-CD3^+^ anti-CD28. Immediately following their initial 24 h growth period, control cells entered the expected rapid proliferative phase during which time they were subject to metabolomic profiling using mass spectroscopy. Wang et al. (203) found that these cells accumulated metabolites during the initial growth phase and then activated glycolysis and directed glucose into the PPP. Concurrently, FAO declined as did the delivery of pyruvate into the TCA cycle. By contrast, OXPHOS and glutaminolysis increased, with a significant amount of the glutamine-associated carbon and nitrogen ultimately being incorporated into α-ketoglutarate and nucleotides, respectively. This latter finding indicated that exogenous glutamine was directed along two distinct pathways, the first being the TCA cycle in which glutamine was converted to glutamate and then to α-ketoglutarate and the second being the purine synthesis pathway in which both the N3 and N6 positions of the purine ring are derived from the glutamine amide moiety. Thus, not unlike the case of Myc-overexpressing fibroblasts discussed earlier (158), T-cell activation was accompanied by increases in both glycolysis and OXPHOS although the source of substrates for the latter pathway shifted from fatty acids and glucose to glutamine. Impaired proliferation was observed when the cells were deprived of either glucose or glutamine or when glycolysis and glutaminolysis, but not FAO, were blocked pharmacologically.

To investigate the molecular basis for the above-described metabolic reprogramming, Wang et al. (203) excised Myc from *myc^flox/flox^* T cells following conditional activation of a 4OHT-inducible CreER transgene and compared their *ex vivo* response to anti-CD3^+^ anti-CD28 activation to the above control *myc^flox/flox^* cells. They noted a severe impairment of both the initial mass accretion (growth) phase and the subsequent expansion phase. *In vivo* testing of these cells following their stimulation with staphylococcal enterotoxin B revealed a blunted response similar to that observed *ex vivo*, with a decrease in both the growth and activation phases. Metabolomic profiling showed that the accumulation of amino acids, nucleotides and lipids in these cells was lower than that measured in similarly activated *myc^flox/flox^* cells. Of note was that *myc^−/−^* cells still activated both ERK and AKT pathways at levels commensurate with those seen in control *myc^flox/flox^* cells. This supported the idea that the observed effects were not a consequence of an inability to respond to signals upstream from Myc but rather to the lack of Myc itself. Further metabolomic inquiry showed that *myc^−/−^* T cells had impaired FAO and glucose flux during both the growth and proliferative phase, accumulated less lactate and directed less glucose-derived carbon into the PPP. Consistent with these findings, the induction of several glycolytic enzymes and glucose transporters were also suppressed as was glutaminolysis. The PKM2 isoform was also less highly induced in *myc^−/−^* T-cells.

## The Role of Glutaminolysis in Metabolic Reprogramming

It has been known since the mid-1950s that both normal and transformed cells share a particular predilection for exogenous glutamine and sometimes even prefer it to glucose as an energy-generating substrate (204–207). Indeed, some tumors have such exaggerated demands for this amino acid that they can deplete host plasma glutamine levels despite its being the most abundant amino acid (208, 209). Recent evidence supports the idea that glycolysis and glutaminolysis cooperatively support high rates of cell proliferation (210, 211).

**Figure 3:**
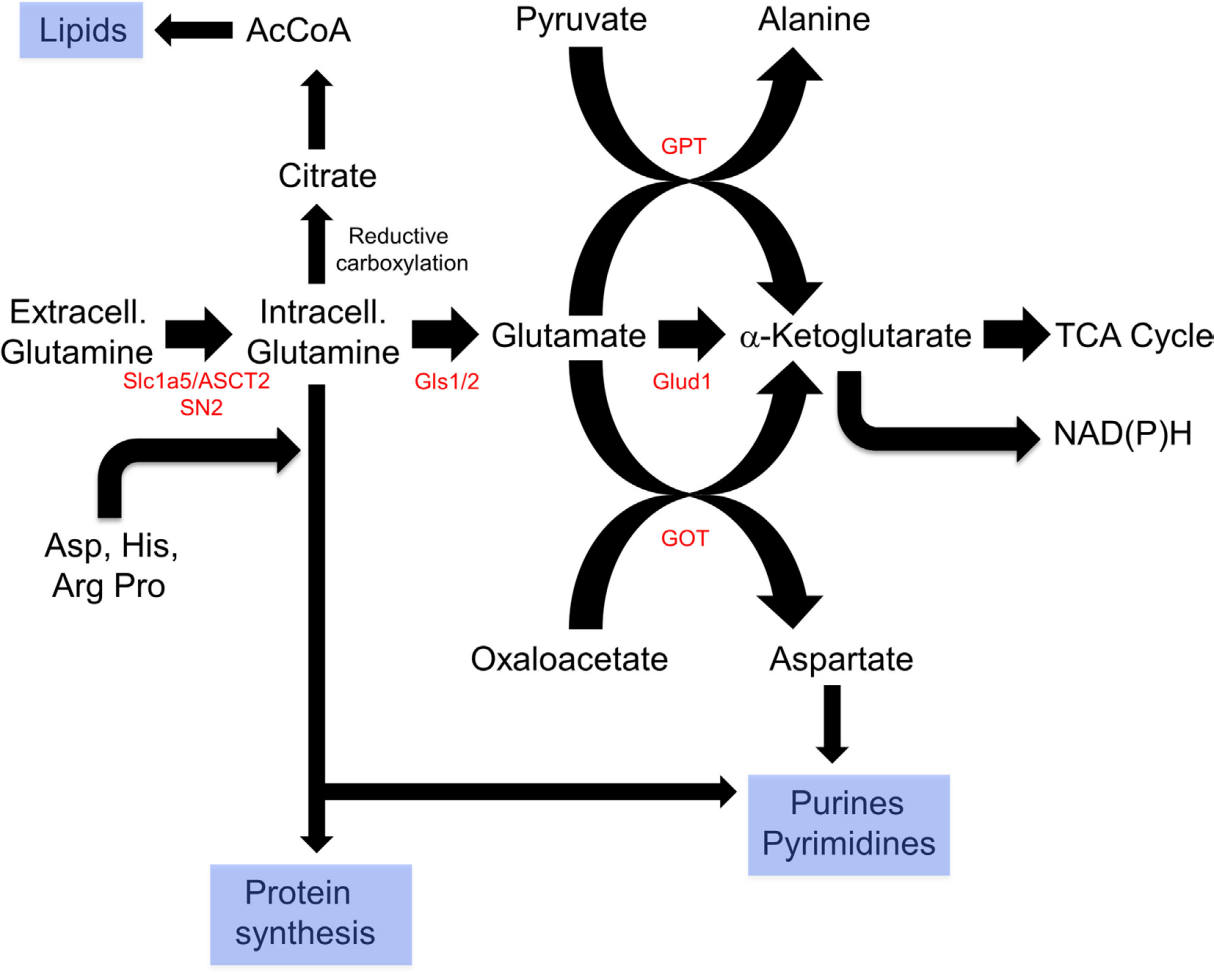
Reprogramming of glutamine metabolism by rapidly proliferating cells. Extracellular glutamine, along with several other amino acids, is delivered to the cytosol by the SLC1A5/ASCT2 or SN2 transporters (212–214). Together, these contribute to *de novo* protein biosynthesis, whereas aspartate is also used in purine and pyrimidine synthetic pathways. In addition, glutamine can undergo reductive carboxylation to supply citrate, which can be converted by ATP citrate lyase to supply cytosolic acetyl-CoA (AcCoA) for *de novo* lipid synthesis (216, 217). Finally, glutamine can be catalyzed by glutamine synthase 1 (Gls1)/2 and then to α-ketoglutarate by the mitochondrial enzyme glutamine dehydrogenase generating a molecule of NADH (or NADPH) in a glycolysis-independent manner. α-Ketoglutarate can also be furnished *via* the action of glutamine pyruvate transaminase (GPT) or glutamate oxaloacetate transaminase (GOT), which utilize pyruvate or oxaloacetate, respectively, in reactions that also yield alanine and aspartic acid. Taken together, glutaminolysis thus coordinates amino acid, lipid, and nucleoside biosynthesis while concurrently providing energy to support these processes and contributing to the anaplerotic substrate supply.

Glutaminolysis offers several advantages that explain its ability to complement and/or replace glucose as an energy source (Figure 3). First, it can be used directly for *de novo* protein synthesis as can the amino acids derived from it including glutamate, proline, histidine, alanine, aspartic acid, and arginine. Second, it may facilitate the uptake of other amino acids, thereby regulating and coordinating their availability for protein synthesis as well (212–214). Third, it serves as the starting point for the biosynthesis of purine nucleosides, thereby linking protein and nucleic acid synthesis. It is noteworthy that the NADPH derived from the diversion of glycolytic substrates into the PPP can also positively impact glutamine uptake and sustain reactions involved in lipid synthesis (215, 216). Fourth, the glutamate dehydrogenase-mediated conversion of glutamate to α-ketoglutarate generates an additional molecule of NADH. This, together with the NADH generated by the α-ketoglutarate dehydrogenase and malate dehydrogenase reactions and the FADH2 generated by the SDH reaction, ensures that nearly normal levels of reducing equivalents can be supplied in a manner that is independent of AcCoA, glycolysis and FAO. Fifth, glutamine-derived α-ketoglutarate can also participate in a reverse carboxylation reaction to furnish citrate for the generation of cytoplasmic AcCoA for use in *de novo* fatty acid and cholesterol biosynthesis (216, 217). Finally, during periods of oxidative stress as commonly occur in many tumors, high levels of ROS can inhibit the aconitase-catalyzed conversion of citrate to isocitrate, thus limiting the supply of glycolytically derived α-ketoglutarate. Glutaminolysis provides a means of overcoming metabolic roadblocks such as this and thereby ensuring a more stable and consistent supply of α-ketoglutarate and the downstream reducing equivalents derived from it (215, 218).

Given the central role of glutaminolysis for sustaining cellular proliferation, coupled with a previous finding that the apoptosis mediated by the absence of glutamine is Myc dependent (219), it was not surprising that Myc regulates this process at several levels. Wise et al. (220) first reported that glioma cells consumed large amounts of glutamine to drive OXPHOS and that the shRNA-mediated suppression of Myc reduced glutamine consumption. They further observed that glioma cells were unable to survive in glutamine-deficient medium, even when supplied with glucose, and that the cell-permeable α-ketoglutarate analog dimethyl-α-ketoglutarate could substitute for glutamine, thereby providing strong evidence that the shunting of exogenous glutamine into the TCA cycle was directly responsible its effect on survival. Moreover, they showed that glycolysis and glutaminolysis, while both Myc regulated, were under distinct forms of control by virtue of the fact that PI3K/Akt signaling regulated the former but not the latter. Myc was subsequently found to bind selectively to E-box-containing promoter regions of the glutamine transporter genes *SLC1A5/ASCT2* and *SN2*. shRNA-mediated suppression of Myc resulted in lowered expression of both transporters’ transcripts and reduced glutamine consumption. Finally, the enforced transient expression of high levels of Myc in MEFs upregulated SLCA15/ASCT2 as well as glutamine synthase 1 (Gls1) and diverted glucose away from its oxidative metabolism by the TCA cycle and into the Warburg-type aerobic glycolysis instead. The mechanism by which Myc upregulated Gls1 appeared to involve increased transcription and/or stabilization of its mRNA.

Gao et al. (221) extended these results by showing that the levels of Gls1 protein in P-493 B cells and PC3 prostate cancer cells varied in direct proportion to the degree of Myc expression. However, and in contrast to Wise et al. (220), this was not true for Gls1 transcript levels leading the investigators to eventually determine that Myc regulated Gls1 at the posttranscriptional level by inhibiting the expression of two microRNAs, miR23a and miR23b (miR23a/b). Both miRNAs were noted to have homology to potential “seed” binding sequences in the Gls1 mRNA 3′-untranslated (3′ UT) region. A luciferase reporter vector containing the Gls1 3′ UTR was shown to be responsive to these miRNAs. Thus, the Myc-mediated upregulation of Gls1is indirect by virtue of its inhibition of at least two miRNAs, which inhibit Gls1 mRNA translation.

In a variation of the above theme, Qing et al. (222), extended these findings to include several human neuroblastoma cell lines and 80 primary human neuroblastomas with varying degrees of N-Myc overexpression. As with the studies of Wise et al. and Gao et al. (221), they found that the cell lines underwent apoptosis in an N-Myc-dependent manner when deprived of glutamine. They also found higher levels of expression of Gls2 (but not Gls1), glutamate oxaloacetate transaminase (GOT2), SLCA15/ASCT2 and several other amino acid transporters that correlated with N-Myc expression levels. Apoptosis in cell lines in response to glutamine deprivation could also be inhibited or delayed by providing the cell-permeable TCA substrate dimethyl α-ketoglutarate in place of glutamine.

Pérez-Escuredo et al. (211) showed that the uptake of ^3^H-glutamine and its utilization by highly oxidative cervical cancer cells was enhanced by lactate, which also accelerated tumor growth. They also showed that Matrigel-embedded tumor cells grown subcutaneously in immunocompromised nu/nu mice in the presence of high local concentrations of extracellular lactate upregulated Slca1A5/ASCT2 and Gls1 at the protein level. shRNA-mediated knockdown of the lactate transporter MCT1 abolished these effects indicating that actual transport of extracellular lactate was mediating the effects on glutaminolysis. The upregulation of Slca1A5/ASCT2 and Gls1 appeared to be mediated by Myc, whose levels were significantly increased by as little as a 6-h exposure to lactate. Further investigation found that the conversion of lactate to pyruvate blocked the activity of prolyl hydroxylases, which are negative regulators of hypoxia-inducible factors (HIFs) 1α and 2α (223). It was suggested that HIF-2α stabilizes Myc *via* its intranuclear binding to Myc-Max heterodimers (224) and indeed, this tripartite interaction was observed in co-immunoprecipitation experiments. Furthermore, the silencing of HIF-2α abolished the upregulation of Myc, Slc1A5/ASCT2 and Gls1. Although HIF-1α was not shown to interact with Myc or Max, it is known to collaborate with Myc to induce the expression of glycolytic genes, thus potentially contributing to the intracellular lactate burden and further stabilizing Myc (224). These effects may have been further aided by the stabilization of HIF-1α by Myc itself (225).

Wang et al. (203) also examined glutamine dependency in the previously mentioned model of normal T-cell activation discussed earlier. They determined that glutamine deprivation resulted in impaired T-cell activation as well as decreased lipid and protein biosynthesis and led to an eventual G_0_/G_1_ arrest without affecting viability.

Although the Warburg effect and glutaminolysis are typically associated with high levels of proliferation (1, 2, 5, 226), they have also been observed in response to hypertrophy in otherwise non-dividing cells. Piao et al. (227), showed that the heart, which relies primarily on glycolysis and FAO for energy, reverts to using glutminolysis and also increases glucose utilization when subject to conditions that induce hypertrophy. Using two different models of right ventricular hypertrophy, they found variable degrees of Myc induction and increased expression of glutamine receptors SLC1A5/ASCT2 and Slc7A5 as well as increases in the mRNAs encoding the Glut1 glucose transporter and hexokinase (HK) 1. Consistent with the former of these findings, the investigators also noted increased ^14^CO_2_ production derived from ^14^C-labeled glutamine. In response to the glutamine antagonist 6-diazo-5-oxo-l-norleucine a decrease in glutaminolysis was noted and was associated with a compensatory increase in glucose oxidation and elevated cardiac output. These studies strongly implicate glutaminolysis as being directly involved in the biomass accumulation that accompanies active proliferation but not in proliferation *per se*.

## Distinct *In Vivo* Metabolic Roles for MYC: Not Always the Same Function in Normal and Neoplastic Tissues

Myc’s unequivocal role in integrating normal mass accretion and proliferative signals with altered metabolism in fibroblasts, myeloid cells, T-cells, and other cell types *in vitro* as discussed earlier contrasts sharply with recent studies in hepatocytes where Myc was found to be entirely dispensable for the long-term regeneration of normal liver parenchyma (228). Several previous studies had indicated that mice with a conditional, hepatocyte-specific knockout of the *myc* gene could regenerate hepatic mass following two-thirds partial hepatectomy (PH) (229–232). Less clear was whether this was achieved as rapidly as occurred in control livers. To some extent, this uncertainty was the consequence of different groups having used different and mostly indirect techniques to measure hepatocyte proliferation and liver regeneration. Further compounding this was the fact that the PH model is a relatively crude and suboptimal way to measure long-term regenerative potential given that the average hepatocyte must divide only about 1.6 times to replace the resected liver mass and that the entire regenerative process is complete within 7–10 days (233). Moreover, as many as 30–40% of the hepatocytes in the regenerating liver remnant remain quiescent following PH, and about the same amount of “regeneration” can be attributed to hypertrophy rather than actual cell division (234, 235). Thus, none of these reports actually addressed the question of whether Myc was necessary to support sustained, long-term hepatocyte proliferation as might occur during the course of normal hepatocyte turnover or repair from chronic injury, both of which are processes of much longer duration (234). It further left open the questions of whether subtle but nonetheless significant differences in regeneration rates might have escaped detection using the PH model and what, in fact, was actually being measured in these other reports.

Edmunds et al. (228) addressed all of these issues by capitalizing on an elegant, robust, and sensitive murine model of Type I hereditary tyrosinemia (236, 237). In these mice, as in humans, inactivation of the fumarylacetoacetate hydrolase (FAH) gene, which encodes the final enzyme in the pathway for tyrosine catabolism, leads to the accumulation of toxic levels of the upstream tyrosine catabolites maleylacetoacetate and fumarylacetoacetate, eventually causing hepatocyte death, fibrosis, and hepatic failure (238). This ultimately fatal outcome can be blocked with the drug 2-(2-nitro-4-trifluoromethylbenzoyl)-1,3-cyclohexanedione (Nitisinone or NTBC), a reversible inhibitor of the enzyme 4-hydroxyphenylpyruvate dioxygenase (HPPD) (239). HPPD is a more proximal enzyme in the pathway and converts the first tyrosine catabolite 4-hydroxyphenylpyruvate to homogenistate. In this way, *fah^−/−^* mice can be maintained in a healthy state simply by providing NTBC in their drinking water and thereby blocking tyrosine catabolism and the accumulation of the deleterious metabolites.

*Fah^−/−^* mice can also be cured by the intrasplenic injection of as few as 10^5^ *fah^+/+^* hepatocytes followed by the intermittent discontinuation and resumption of NTBC (236, 237). Animals initially lose weight as they accumulate the toxic tyrosine catabolites. However, as endogenous *fah^−/−^* hepatocytes are gradually replaced by the *fah^+/+^* donor population over 4–5 months, the recipient mice are eventually rendered NTBC-free and their livers are comprised of 50–80% donor hepatocytes (228, 236, 237, 240). As a way of monitoring hepatocyte proliferative potential, this model offers several advantages over PH. First, because the donor cells must divide 50- to 100-fold during recipient liver repopulation, they undergo many more population doublings than do post-PH hepatocytes, thereby providing a more demanding and long-term replicative challenge. Second, donor hepatocytes from different sources can be used in “competitive” repopulation assays, analogous to those used for decades in bone marrow transplantation studies (241, 242). Provided that the recipient and donor populations can be distinguished, the ultimate contribution of each to the steady-state transplanted liver can be assessed with exquisite precision and even quite small deviations from the input donor ratios can be easily quantified (228, 240). Third, if so desired, the input ratio of the competing donor populations can be varied to reveal even more dramatic differences in regenerative potential. Finally, because the competing donor populations replicate in identical environments, differences in regeneration rates can be ascertained with many fewer animals than are required with PH-based experiments.

Edmunds et al. (228) exploited the FAH model to assess the regenerative capacities of mixed *fah^+/+^* populations of *myc^+/+^* and *myc^−/−^* hepatocytes. Surprisingly, the ratio of the two donor hepatocyte populations recovered from the fully reconstituted recipient livers more than 4 months after their co-transplantation was identical to that of the input donor populations. Thus, even under the most demanding of circumstances, Myc’s absence did not impair the long-term regenerative potential of hepatocytes in this particular model.

In addition to being at odds with the above-discussed role for Myc in the proliferation of fibroblasts and T-cells and numerous other cell types (154, 158, 191, 196, 203), these results also differ from studies in *Drosophila* and some cancer lines showing that cells expressing higher levels of Myc tend to outcompete those with low levels (243–245). Similarly, the conditional deletion of *myc* or its dominant-negative inhibition in intestinal crypt cells or bone marrow cells is associated with severe proliferative defects although these may be ameliorated over time (49, 246–248). Thus, the elimination of endogenous Myc seems to have highly variable and tissue-specific effects, with liver representing an atypical although perhaps not unique example.

Ultimately, while the metabolic consequences of endogenous Myc loss are tissue specific and variable, it seems reasonable to conclude that, in most cases, Myc is responsible for maintaining context-appropriate levels of ATP and anabolic substrates by regulating the uptake and oxidation of nutrients that furnish glycolysis and the TCA cycle. In Myc’s absence, as noted above, many tissue types appear to adapt to the associated nutrient and energy deficits *via* various strategies that include variable reductions in cell mass, proliferative rate and anabolic activity (49, 158, 180, 228, 249). Interestingly, while Edmunds et al. (228) did not observe any significant differences in cell size, ATP levels or AMPK phosphorylation in the livers of mice following transplantation with *myc^+/+^* or *myc^−/−^* hepatocytes, these studies were performed on hepatocytes that had already re-populated the liver and reached a non-proliferating equilibrium state. It is certainly possible that more profound energy deficits might have been observed had the actual proliferating population been assessed at an earlier time following transplant. Nonetheless, these studies clearly demonstrated that *myc^−/−^* hepatocytes remain as fully capable as their wild-type counterparts at contributing to the long-term repopulation of the liver irrespective of whatever defects they may harbor.

Although *myc^−/−^* hepatocytes demonstrated no obvious proliferative impairment in the above-described repopulation assay, they nevertheless showed several abnormalities that were evident even prior to transplantation (228). First, despite body weights identical to those of *myc^+/+^* mice, juvenile mice with hepatocyte-specific deletion of *myc* had smaller livers, consistent with a previous observation that *myc* hypomorph mice tend to have smaller numbers of otherwise normal-sized cells in some organs, including liver (249, 250). At first glance, this would seem to be inconsistent with fact that *myc^+/+^* and *myc^−/−^* hepatocytes competed equally in repopulation studies (228). However, it is possible that the requirements for Myc in the developing liver versus the fully developed liver are different. There may thus exist a phase early in development, but not beyond, during which Myc is required for hepatocyte expansion. Alternatively, the smaller size of *myc^−/−^* livers may more reflect an unappreciated role of Myc in regulating organ size (251, 252) than in limiting the proliferative potential of its individual constituent cells, which is what is measured in hepatocyte transplant studies.

In contrast to the above findings, adult *myc^−/−^* livers actually weighed *more* than *myc^+/+^* livers. The former possessed a significantly higher neutral lipid and triglyceride content, which likely accounted for their increased mass (228). This implied either that *myc^−/−^* livers take up and store greater amounts of these lipids and/or utilize less of them. Arguing against the latter point was the finding that *myc^−/−^* livers showed variable but significant increases in FAO. This suggested that, in the absence of Myc, hepatocytes both take up and utilize more fatty acids, with the former process outpacing the latter, eventually culminating in an increased storage pool, not unlike that seen in *myc^−/−^* fibroblasts or following short-term pharmacologic Myc or N-Myc inhibition in other cell types (191, 196, 228).

Edmunds et al. (228) studied the structure and function of isolated mitochondria from *myc^+/+^* and *myc^−/−^* livers using BNGE and noted no obvious differences in the stoichiometries of the protein subunits of ETC Complexes I–IV or the ATP synthase (Complex V). Mass spectroscopic quantification of over 400 mitochondrial proteins, including all 93 subunits of the ETC, also showed no significant quantitative differences between the two groups. However, Complex I and Complex II activities were modestly but significantly reduced in mitochondria from *myc^−/−^* livers in response to ADP and succinate whereas the activity of Complex V was increased by about the same amount. Coupled with the finding that ATP levels in *myc^+/+^* and *myc^−/−^* livers were comparable, this suggested that the loss in ETC function in *myc^−/−^* livers was compensated for by a more efficient generation of ATP *via* Complex V and/or by the more inherently efficient process of FAO (253).

**Figure 4:**
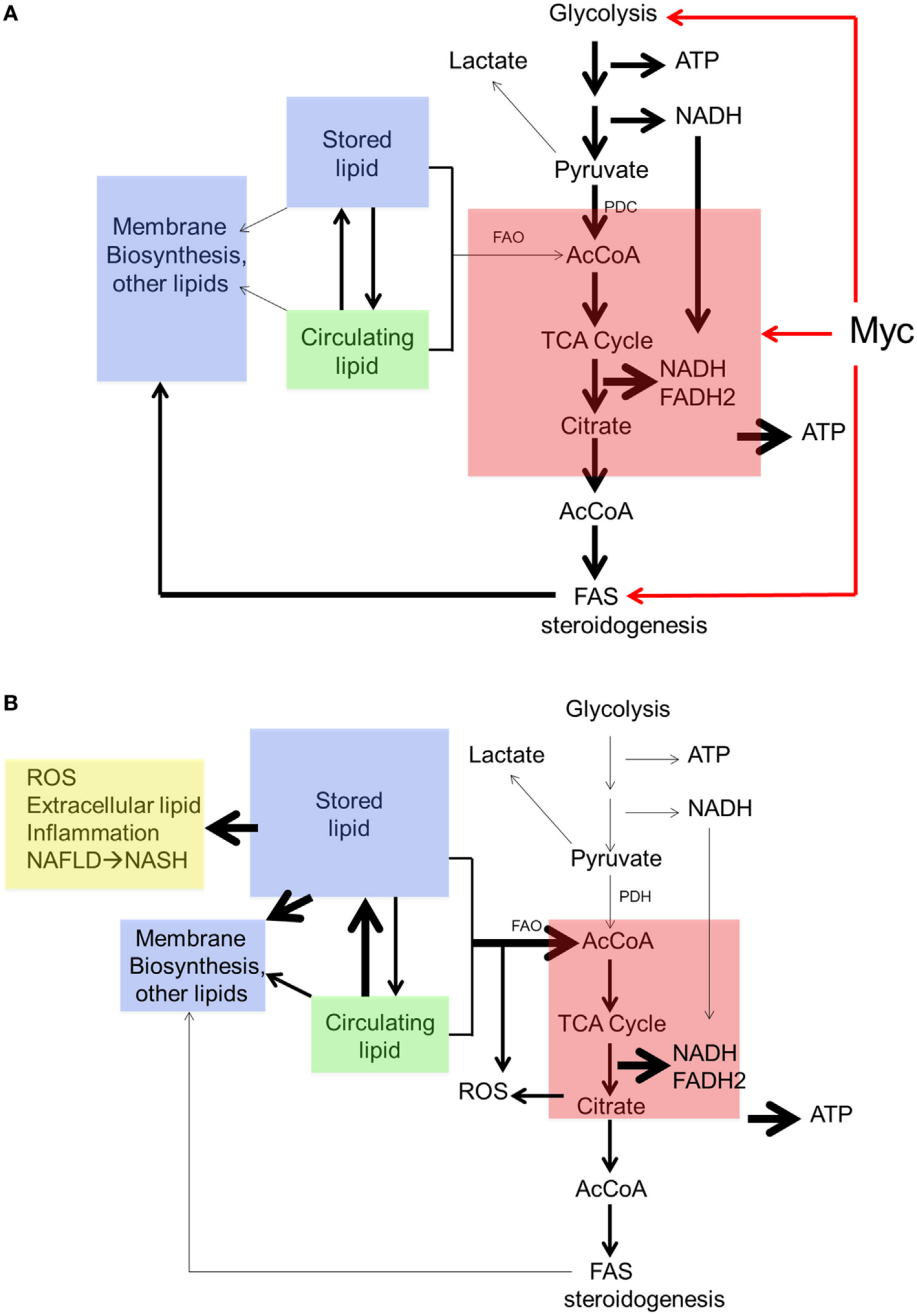
Regulation of neutral lipid accumulation in *myc^−/−^* hepatocytes is both Myc dependent and Myc independent. **(A)** In *myc^+/+^* hepatocytes, the pathways indicated by red arrows are regulated both by Myc and Myc-independent factors. Stored lipid and circulating lipid are maintained in equilibrium. Stored lipid contributes minimally to energy generation (*via* FAO) or the synthesis of new membranes or other lipids when ATP and acetyl-CoA (AcCoA) are derived *via* glycolysis. Under these conditions, lipids such as fatty acids and sterols are largely synthesized from cytoplasmic AcCoA that originates from TCA cycle-derived citrate. **(B)** In *myc^−/−^* hepatocytes, basal rates of glycolysis, oxidative phosphorylation, and fatty acid synthesis are depressed thereby allowing for a compensatory increase in FAO. This allows basal levels of ATP and AcCoA to be sustained to meet energetic needs. New membrane synthesis thus becomes more reliant upon the uptake of exogenous lipids, which accumulate in the form of neutral lipids, leading to mitochondrial dysfunction and reactive oxygen species (ROS) generation (254–256). Additional ROS are generated as a result of the accumulation of intracellular and extracellular neutral lipid (257–259).

Collectively, these findings suggest a form of metabolic reprogramming by the *myc^−/−^* liver only partially resembling that seen in *myc^−/−^* fibroblasts, which may reflect the different tissues under consideration as well as their different proliferative rates. For example, ATP and AcCoA levels were markedly diminished in *myc^−/−^* fibroblasts but were maintained at normal levels in *myc^−/−^* livers (158, 228) (Figure 4). This appears to have been due to the employment of FAO as an alternative energy source by hepatocytes as well as to an increase in neutral lipid accumulation that has been previously reported in *myc^−/−^* fibroblasts, in hematopoietic cells following short-term inhibition of Myc, and in neuroblastomas following treatment with small molecule Myc inhibitors (158, 191, 196). The neutral lipid accumulation by *myc^−/−^* hepatocytes was even more striking following their transplantation into *fah^−/−^* recipient mice. Oil Red O staining of fully reconstituted livers confirmed their significantly higher triglyceride content as well as larger and more numerous neutral lipid droplets (228). Indeed, the lipid within these livers was so abundant that much of it was extracellular. This was likely responsible for the significant inflammatory cell infiltrate that was observed as well as for the upregulation of numerous transcripts involved in acute and chronic inflammation, leukocyte signaling and fibrosis. Immunohistochemical staining for 4-hydroxynonenal, a by-product of ROS-mediated lipid peroxidation, was also detected as was evidence for dysregulated mitochondrial structure and function as previously reported in mice maintained on high fat diets (254–256). Other findings included the downregulation of 19 of the 44 transcripts encoding subunits of Complex I and seven of the 20 transcripts encoding subunits of Complex V. In the latter group, 10 of the remaining 13 transcripts were upregulated. Taken together, these studies suggested that the loss of Myc expression in resting hepatocytes is initially associated with the gradual accumulation of neutral lipid, not unlike that seen in non-alcoholic fatty liver disease (NAFLD) in association with ETC dysfunction (257, 258). When coupled with the metabolic stress imposed by regeneration-associated proliferation and chronic inflammation, this progressed to a phenotype closely resembling non-alcoholic steatohepatitis (NASH), a long-term consequence of NAFLD that is associated with high levels of oxidative stress, inflammation, fibrosis and eventual hepatic failure (257, 259). It is tempting to speculate that the loss of Myc in these hepatocytes, particularly during times of proliferation and high energy demand, leads to an attenuated glycolytic response and the dysregulation of mitochondrial structure and function. The resulting energy depletion and mitochondrial stress is accompanied by an increased reliance on FAO coupled with unbalanced fatty acid uptake and storage, intracellular ROS generation, inflammation, and long-term parenchymal damage that mimics NAFLD and NASH (228).

The above findings do not provide simple explanations for how endogenous Myc affects metabolism during normal growth. They do suggest, however, that these changes occur at multiple levels and that the compensation by *myc^−/−^* hepatocytes ultimately affects neither ATP generation, AcCoA levels nor proliferation. It remains to be determined precisely how ATP levels in *myc^−/−^* livers are maintained but it appears to be dependent on several different pathways including an increased efficiency of ATP production as suggested by the Complex V assays and the provision of alternate sources of AcCoA mainly from enhanced FAO. An alternative source might originate with increased glutaminolysis, which could conceivable circumvent the need to rely on glycolysis- or FAO-derived AcCoA by providing reducing equivalents in the form of both NADH and FADH2. Whatever the cumulative corrective mechanisms are that help to normalize hepatocyte energy generation and proliferative function, they come at considerable metabolic cost in the form of increasingly severe lipid storage and utilization defects.

Metabolic changes have also been studied in *myc^−/−^* hepatocytes in response to oncogenic signaling following the induction of hepatoblastoma (HB). HB is the most common pediatric liver cancer, almost invariably arising in children under the age of 3 years and is associated with somatic mutations of the β-catenin gene in >80% of cases (260, 261). A useful mouse model of HB has recently been described in which tumors arise with nearly 100% penetrance following the hydrodynamic tail vein injection-mediated delivery of “Sleeping Beauty” plasmids (262, 263) encoding patient-derived mutant forms of β-catenin and yes-associated protein (YAP) (264–266). A major transcriptional target for β-catenin is Myc (267) and, not unexpectedly, Myc is among the most highly upregulated genes in HBs (Monga and Prochownik, unpublished data). Given that Myc plays no role in normal hepatocyte repopulation as discussed earlier (228), it was of interest to determine whether Myc is needed to support the more rapid growth and increased metabolic demands of HBs. Indeed, Wang et al. (125) found that, although tumors arose in *myc^−/−^* livers at the same frequency as they did in *myc^+/+^* livers, their growth rates were significantly impaired. Thus, while Myc was dispensable for normal hepatocyte proliferation and for the induction of HBs, it was clearly required for determining the rate of tumor growth (125, 228).

To understand the basis for Myc’s selective role in HB proliferation (228), the metabolic properties of *myc^+/+^* and *myc^−/−^* HBs were compared. An initial assessment of mitochondrial function showed both types of HBs had lower OCRs and Complex II activity compared with corresponding livers. However, less suppression of these activities was seen in *myc^−/−^* HBs. It is likely that this reflected their slower growth rates and thus a reduced tendency to rely on Warburg-type aerobic glycolysis for energy generation. Consistent with this, glycolytic transcripts as a group were upregulated in *myc^+/+^* HBs to a significantly greater extent than they were in *myc^−/−^* HBs (11.2-fold versus 8.9-fold, *P* < 0.0004). The presumptive lower glycolytic rate of the latter likely accounted for their lower AcCoA levels. Despite these differences, both tumor types showed elevated levels of ATP and a downregulation of phosphoAMPK relative to their respective livers. This suggested that, despite differences in their metabolic activities, the slower growth rates of *myc^−/−^* HBs were not the result of any obvious energy deficit. On the other hand, it is conceivable that *myc^−/−^* HBs were energetically constrained and that they maintained a rate of growth that was compatible with these limitations while still allowing normal levels of ATP to be maintained. The presumptive reduced rate of glycolysis by *myc^−/−^* HBs may also have restrained the supply of anabolic precursors necessary for sustaining rapid tumor growth. In addition, these tumors were also less able than *myc^+/+^* tumors to upregulate the group of transcripts encoding the ~80 ribosomal protein genes (3.6-fold versus 5.2-fold, *P* < 10^−4^) (125). Thus, rather than being severely deprived of energy, as in the case for *myc^−/−^* fibroblasts, *myc^−/−^* HBs may instead be deprived of anabolic precursors and the ability to increase protein synthesis rates, despite adequate energy supplies. Protein synthesis rates and ribosomal protein content are known to be rate-limiting factors in the growth of many cancers (108, 268).

In further pursuit of an explanation for the overall downregulation of OXPHOS by tumors, Edmunds et al. (228) quantified mtDNA using qPCR to amplify two distinct regions of the mitochondrial genome. They documented a ~60–80% reduced mitochondrial mass in both *myc^+/+^* and *myc^−/−^* tumors, thus providing a structural explanation for the Warburg effect. Results published at about the same time by Reznick et al. (269) showed a similar loss of mtDNA from a wide variety of human cancers whose genomic data had been compiled in *The Cancer Genome Atlas* (https://cancergenome.nih.gov).

In HBs arising in *myc^+/+^* and *myc^−/−^* livers, eight of the 14 most deregulated pathways identified by Ingenuity Pathway Analysis involved lipid biosynthesis. Importantly, transcripts encoding several key enzymes involved in fatty acid synthesis (FAS) such as ATP citrate lyase (ACLY), fatty acid synthase, and AcCoA carboxylase were markedly upregulated, although not in a Myc-dependent manner. By contrast, transcripts encoding enzymes involved in FAO such as trifunctional protein, carnitine palmitoyltransferase-2 and very long-chain acyl-CoA dehydrogenase were markedly downregulated. Indeed, even transcripts whose encoded enzymes participate in peroxisomal FAO such as the fatty acid transporter ATP-binding cassette-D3, peroxisomal biofunctionalized protein, and acyl-CoA oxidase-1 were downregulated. Functional assays of the FAO pathway were consistent with these findings and showed marked downregulation of FAO activity in both *myc^+/+^* and *myc^−/−^* tumors (125). Taken together, these studies imply that, unlike the case in fibroblasts, where the loss of Myc leads to a near cessation of both glycolysis and OXPHOS and an upregulation of FAO as an alternate energy source, in HBs, glycolysis and OXPHOS are much less affected and rely less on FAO to supply energy.

## Metabolic Links Between Normal and Neoplastic States

As discussed at length above, proliferating tumor cells require a continual supply of the basic cellular building blocks consisting of amino acids, nucleic acids, and lipids. In this regard, tumor cells are not very different from the progenitor cells that give rise to our various organs during embryogenesis, or the regenerative stem cells now known to reside within many of our adult tissues. Furthermore, differentiated cells may dedifferentiate and then proliferate to repair an injury, followed by re-differentiation. The metabolic similarities between these different types of proliferating cells are striking: all rely heavily upon glucose and glutamine to meet their steep anabolic needs (270–272). Not coincidentally, Myc maintains stem cell pluripotency and self-renewal, perhaps through its effects on driving glucose/glutamine uptake and metabolism (273). On the other end of the spectrum are non-proliferative, differentiated cells that are often metabolically reliant upon FAO. Several fetal tissues that rely upon glucose during growth and development undergo a metabolic switch to FAO after birth. The best-known example of this is the heart (274) and we have made similar observations in human kidney (Goetzman, unpublished data). Senescence is also characterized by a switch to FAO while lipid synthesis is turned off (275, 276). With some notable exceptions (i.e., prostate cancer) most cancers are glucose/glutamine dependent and not FAO dependent (277, 278).

The glucose/glutamine versus FAO dichotomy is maintained in both normal and neoplastic tissues by a key metabolic phenomenon called the Randle cycle, sometimes simply referred to as the glucose–fatty acid cycle. The Randle cycle refers to the reciprocal nature of mitochondrial rates of pyruvate oxidation and FAO (279, 280). The Randle cycle centers on pyruvate dehydrogenase (PDH) and carnitine palmitoyltransferase 1 (CPT1), which are the rate-limiting enzymes of pyruvate oxidation and FAO, respectively. When glucose is abundant and glycolysis is high, pyruvate concentrations rise and inhibit pyruvate dehydrogenase kinase (PDK1), the kinase responsible for phosphorylating and silencing PDH. PDK1 inhibition activates PDH and increases mitochondrial pyruvate oxidation to generate AcCoA and NADH. Increases in intramitochondrial AcCoA and NADH have two consequences for FAO. First, the intramitochondrial FAO machinery is directly inhibited by high NADH/NAD^+^ and AcCoA/CoA ratios (281). Second, the entry of pyruvate-derived AcCoA into the TCA cycle produces citrate, which is exported from mitochondria to the cytosol where it is reconverted to AcCoA *via* the action of ACYL. Cytoplasmic AcCoA is then converted to malonyl-CoA by AcCoA carboxylase (ACC). Malonyl-CoA in turn is a potent inhibitor of CPT1. CPT1 is the gatekeeper for FAO *via* its role in regulating fatty acid transport across the mitochondrial membrane; thus, the result of sustained PDH flux is the sequestration of fatty acids in the cytosol, away from the degradative FAO machinery. Cytosolic fatty acids are then either converted to triglyceride droplets to fuel the cell should glucose become unavailable, or in the case of proliferating cells, incorporated into phospholipid acyl-chains for synthesizing new membranes. The same malonyl-CoA which silences CPT1 also feeds into the *de novo* FAS pathway to further support the anabolic needs of the cell (282). Because of this cycle, the rate of PDH flux has been shown to highly correlate with lipogenesis in many cell types. In the liver, for example, insulin increases PDH activity and lipogenesis from glucose-derived AcCoA, while starvation rapidly inhibits PDH activity and thus limits glucose-derived lipogenesis (283).

**Figure 5:**
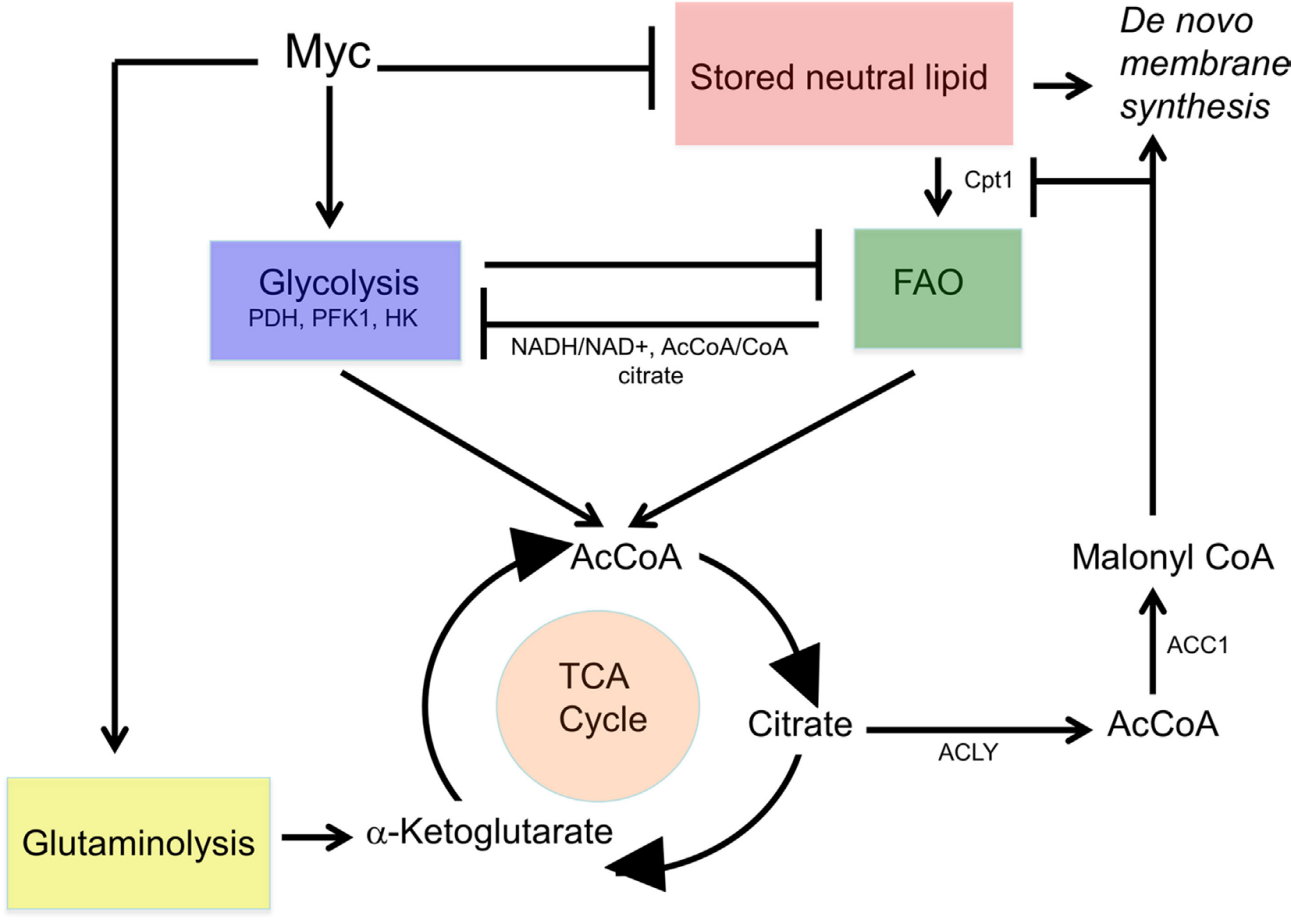
Co-regulation of glycolysis and FAO. Starvation or glucose deprivation mobilizes lipolytic pathways and upregulates FAO to provide an alternate source of acetyl-CoA (AcCoA) and maintain oxidative phosphorylation. More so than glycolysis, FAO increases NADH/NAD^+^ and AcCoA/coenzyme A ratios as well as cytoplasmic citrate thereby inhibiting pyruvate dehydrogenase (PDH), phosphofructokinase, hexokinase (HK), and glucose uptake (284–286). By contrast, glucose utilization by the TCA cycle tends to produce a greater buildup of citrate-derived, cytoplasmic coenzyme A (CoA) and malonyl-CoA, which exerts potent negative control over Cpt1.

The Randle cycle also works in the opposite direction, i.e., high rates of FAO suppress glucose utilization. In the same way that glucose-derived AcCoA and NADH inhibits the FAO machinery, FAO-derived AcCoA and NADH potently inhibit PDH *via* activation of PDK1. There is also some evidence that fatty acid-derived citrate exported to the cytosol can suppress glycolysis at the level of phosphofructokinase-1 (284–286). The resulting accumulation of glucose-6-phosphate feeds back to inhibit hexokinase (HK) and glucose uptake (Figure 5) (286). Overall, the net result of these various points of negative feedback control are a downregulation of glucose utilization by FAO. A key theme of the Randle cycle is that lipogenesis sides with glucose oxidation, and that cells cannot simultaneously conduct FAO and FAS. This is because activation of FAS, by virtue of cytosolic conversion of citrate to AcCoA to malonyl-CoA, would inhibit FAO at the level of CPT1. This maybe the reason why proliferating cells, be they cancerous or not, suppress FAO such that they can drive their absolute requirement for FAS and membrane synthesis. Likewise, it explains how terminally differentiated and senescent cells are able to sustain high rates of FAO for their energetic needs. In short, because of FAO suppression of glucose-derived FAS, it is difficult for cells to proliferate while conducting a high rate of FAO. Exogenous fatty acids can be incorporated into membranes directly, but in FAO-dependent cells the phospholipid synthesis pathways would be in continual competition with mitochondria for incoming exogenous fatty acids.

The Randle cycle and connection between glucose utilization and FAS would suggest that tumors would tend to have low FAO and high FAS. Indeed, this has been observed in many cancers, and inhibition of the FAS pathway has been shown to slow cancer growth in many instances (287, 288). In the HB mouse model discussed at length above, FAO was found to be markedly lower than in normal control liver while PDH activity was increased several-fold (240). This occurred both in the presence and absence of Myc suggesting that there are multiple mechanisms responsible for driving the glucose–fatty acid cycle in tumors. A similar reciprocal relationship between FAO and PDH activity was also seen in a model of hepatocellular carcinoma induced by the deregulated, doxycycline-regulated induction of Myc (126). Of course, as is true with all aspects of cancer metabolism, there have also been multiple studies demonstrating cancers with the opposite metabolic phenotype, i.e., high FAO and low PDH activities (289–294). It is interesting to note that prostate cancer, the best-characterized example of a cancer with high FAO, is well known for its typically slow growth rate. Some studies have shown that maximizing pyruvate flux through PDH by inhibiting PDK with dichloroacetate can sensitize cells to other chemotherapies, presumably by promoting mitochondrially driven apoptosis (295). As with many metabolic pathways, too much of any one thing may lead to cell death. In the case of PDH, it has been shown in normal liver that most PDH capacity is not being used, even under fed, insulin-stimulated conditions (283). Only about 10% of the PDH in fed rodent liver is in the active (unphosphorylated) state; acute insulin exposure doubles this to 20%. Dichloroacetate increased the active state to 100%. Therefore, it remains possible that either supraphysiological activation or inhibition of PDH may be beneficial in crippling cancer growth, with the usual caveat that it depends upon the cancer being treated.

**Figure 6:**
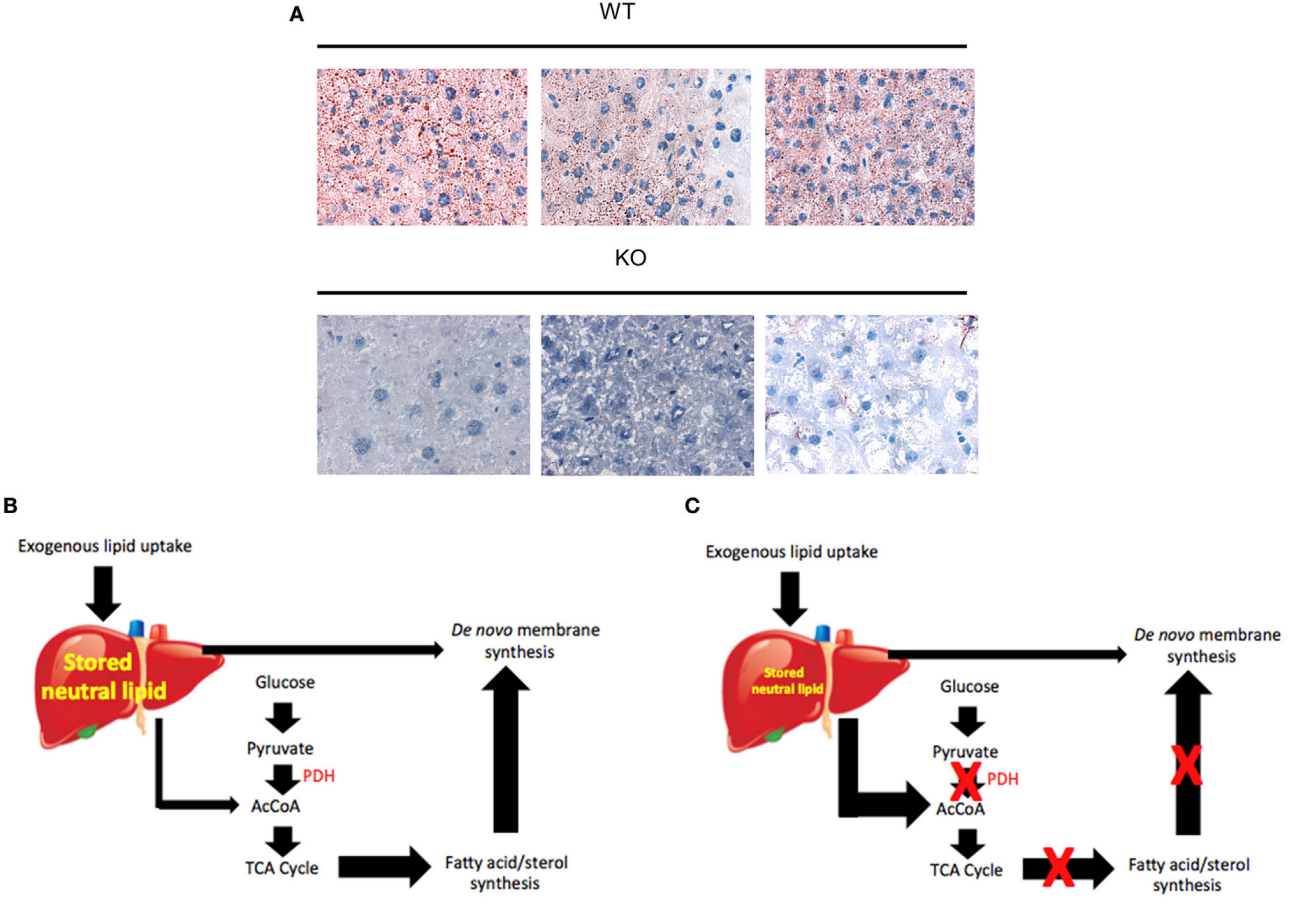
Differential utilization of neutral lipid stores in the absence of *pdha1*. **(A)** Oil Red O-stained liver sections from 6- to 8-week-old *pdha1* WT and KO mice showing the latter to be nearly devoid of neutral lipid. Each panel is from a different animal. All sections are 40× magnification. **(B)** Lipid metabolism in the normal [pyruvate dehydrogenase (PDH)-WT] liver where there exists a balance between exogenous lipid uptake, storage, and utilization. The latter consists of direct incorporation of stored lipids into new membranes and FAO. Both of these occur at low levels because they compete with glycolysis to supply acetyl-CoA (AcCoA). The AcCoA derived from glycolysis is used as a substrate for the energy-generating TCA cycle and the energy-depleting synthesis of *de novo* membrane. **(C)** Lipid metabolism in PDH KO livers. The activation of FAO provides an alternate source of AcCoA when it cannot otherwise be supplied by glycolysis. Although this route is sufficient to serve as an energy-generating substrate, it is insufficient to allow for *de novo* fatty acid and sterol synthesis. Thus, stored hepatic lipid is also utilized directly for this purpose. The net result is the depletion of stored neutral lipid reserves.

With regard to liver cancers, the commonality of high PDH activity in both HBs and HCC (125, 240) led to the hypothesis that eliminating PDH may slow tumor growth. First, Jackson et al. examined the consequences of conditional inactivation of the catalytic PDHA1 subunit on normal hepatocyte proliferation in murine liver (240). Using the above-described competitive hepatocyte re-population assay, it was determined that wild-type and PDH knockout hepatocytes contributed equally to the long-term reconstitution of *fah^−/−^* recipient livers. Thus, despite the severing of the direct line of communication between glycolysis and the TCA cycle, and between glucose utilization and FAS, proliferation remained unaffected. That PDH is profoundly linked to lipogenesis was demonstrated by the striking loss of lipid stores in PDH KO liver (Figure 6). Based on this observation it is tempting to speculate that PDH inhibition could represent a novel therapeutic approach for the treatment of NAFLD. What remains to be determined is the magnitude of PDH suppression needed to achieve this outcome, the length of time PDH suppression must continue, and how rapidly lipid re-accumulates following its reactivation.

Two approaches were next taken to ascertain how inactivation of PDH altered the properties of HBs induced by mutant β-catenin + YAP (125, 240). In the first approach, both the resultant tumors and the adjacent, non-transformed parenchyma were rendered PDH-negative (“pan KO”), whereas in the second, only tumors and not the adjacent non-transformed liver tissue were rendered PDH-negative (“restricted KO”). Survival of the pan KO tumor group did not differ significantly from that of the control group with normal levels of PDH expression in all tissues whereas survival of restricted KO tumor group was significantly prolonged (mean survival ~125 versus 90 days). Further investigation showed that pan KO HBs tended to be smaller than control tumors despite the similar survival of the two groups. By contrast, HBs from the long-surviving restricted KO group were about the same size as control tumors. These apparent discrepancies were ultimately resolved by showing that mice in the pan KO group had a severe lactic acidosis at the time of their demise. It was concluded that, in the face of the absence of PDH activity in pan KO hepatocytes, HB-derived lactate could not be metabolized back to pyruvate by the untransformed hepatocytes as it could by the PDH-expressing hepatocytes of restricted KO mice. The demise of the pan KO animals was thus due to a combination of tumor burden and lactic acidosis. The normal respiratory compensation that attempts to correct metabolic acidosis was almost certainly compromised in these mice as well due to the restricted diaphragmatic mobility imposed by the tumor burden.

The most notable metabolic defect in PDH KO tumors was a *ca*. 80% reduction in total AcCoA levels. Surprisingly, only a slight, statistically non-significant increase in compensatory FAO was observed (240). Importantly, while the Randle cycle shows that driving one pathway will limit the other, these studies suggest that directly limiting one pathway does not necessarily stimulate the other (i.e., eliminating PDH may not necessarily stimulate high FAO). Interestingly and unexpectedly, the PDH KO studies indicated that despite the profound inability to maintain normal levels of AcCoA, KO hepatocytes were able to sustain normal or near-normal rates of growth even following their transformation into highly proliferative tumor cells. It is possible that reductive carboxylation of glutamine, which was not examined, could supply just enough cytosolic AcCoA to drive the fatty acid synthesis needed to supply lipids for new membranes. In this scenario, the minimal induction of compensatory FAO might be explained by glutamine-derived malonyl-CoA, which would limit the capacity for FAO by inhibition of CPT1. In short, this series of studies with PDH KO mice highlight the amazing metabolic plasticity of both normal and neoplastic livers, and stress the difficulty of targeting metabolic pathways to treat cancers.

## Therapeutic Strategies Aimed at Targeting MYC and its Effectors

Myc has long been viewed as an exciting but challenging oncologic target (51, 52, 296). The enthusiasm stems from the fact that Myc is among the most commonly deregulated oncoproteins in all of cancer (24, 51, 52, 97). Even in those neoplasms where Myc deregulation is not immediately appreciable, other oncogenic signaling pathways that *are* deregulated invariably converge upon Myc which then carries out their transcriptional bidding. In these cases, inhibiting Myc still leads to cell cycle arrest, the collapse of ATP production and apoptosis (50, 191). Unfortunately, Myc’s lack of enzymatic activity or prominent structural features makes it extremely difficult to target with small molecules (51, 52, 296). The means by which Myc inhibition has been attempted and descriptions of the successes and (mostly) failures along the way have been recently reviewed (297). These attempts included strategies such as altering the structure of Myc DNA-binding sites, inhibiting the structure and/or function of Myc-Max heterodimers, and inhibiting Myc’s engagement with and activation of the transcriptional activation machinery (297). A second challenge to inhibiting Myc is that the plethora of target genes whose expression is altered, either directly or indirectly, in response to Myc deregulation includes many that are transforming in their own right. Thus, an obvious alternate strategy, such as targeting one of these rather than the intimidating Myc itself, is likely to fail because of their sheer number and oncogenic functional redundancy. A final challenge is that many of the early Myc inhibitors that have been described to date, even those which function well *in vitro*, have demonstrated disappointing therapeutic efficacy *in vivo* due to poor tissue penetration, low potency, or rapid metabolism (51, 52, 296, 297).

While these issues are clearly important when considering the targeting of oncogenic pathways, some become less so when contemplating the employment of inhibitors in non-neoplastic settings where Myc is a potential target that may not require systemic inhibition. For example, the intimal hyperplasia that accompanies atherosclerotic stenosis has long been known to be accompanied by and to be dependent upon Myc overexpression (52, 298, 299). We have proposed that drug-eluting stents, which released Myc inhibitors continuously and over short distances, would provide a means by which high-local compound concentrations could be achieved thus overcoming some of the pharmacologic shortcomings that plague their systemic delivery (52). Myc antisense oligonucleotides have shown efficacy is this regard even though their ability to reduce Myc levels has been somewhat modest (300, 301).

Non-neoplastic settings also now permit the consideration of downstream Myc effectors that would not otherwise represent tractable targets due to their neoplastic redundancy as mentioned earlier. An excellent example of this is PDH, which is neither an oncoprotein nor even a direct Myc target although it is dramatically upregulated and activated in response to activation of the Myc pathway (Figure 6) (125, 126, 240). Inhibiting PDH *via* more traditional approaches than are available for Myc could prove to be of enormous therapeutic benefit for the treatment of NAFLD, a common condition associated with considerable non-neoplastic morbidity and mortality as well being a significant predisposing factor for the development of cancer (257). Time will tell whether the inhibition of Myc and its partners in crime will be of greater therapeutic benefit for cancer, for metabolic disorders, or for those diseases harboring overlapping features of both.

## Author Contributions

This review manuscript was written and edited by EP and EG. EP designed and prepared the figures.

## Conflict of Interest Statement

The authors declare that the research was conducted in the absence of any commercial or financial relationships that could be construed as a potential conflict of interest.
